# COVID-19 inpatient mortality in Brazil from 2020 to 2022: a cross-sectional overview study based on secondary data

**DOI:** 10.1186/s12939-023-02037-8

**Published:** 2023-11-17

**Authors:** Margareth Crisóstomo Portela, Mônica Martins, Sheyla Maria Lemos Lima, Carla Lourenço Tavares de Andrade, Claudia Cristina de Aguiar Pereira

**Affiliations:** Department of Health Administration and Planning, Sergio Arouca National School of Public Health, Oswaldo Cruz Foundation, Rio de Janeiro, RJ Brazil

**Keywords:** SUS, COVID-19, Inpatient healthcare, Inpatient mortality, Inequities, Brazilian health system

## Abstract

**Background:**

In Brazil, the COVID-19 pandemic found the universal and public Unified Health System (SUS) with problems accumulated over time, due, among other reasons, to low investments, and disparities in resource distribution. The preparedness and response of the healthcare system, involving the SUS and a private sector, was affected by large socioeconomic and healthcare access inequities. This work was aimed at offering an overview of COVID-19 inpatient mortality during the pandemic in Brazil, exploring factors associated with its variations and, specifically, differences across public, private (for-profit) and philanthropic (private non-profit) inpatient healthcare units, providers, and non-providers of services to the SUS.

**Methods:**

This cross-sectional study used public secondary data. The main data source was the SIVEP-Gripe, which comprises data on severe acute respiratory illness records prospectively collected. We also employed the National Record of Health Establishments, the SUS’ Hospitalization Information System and municipalities' data from IBGE. We considered adult COVID-19 hospitalizations registered in SIVEP-Gripe from February 2020 to December 2022 in inpatient healthcare units with a minimum of 100 cases in the period. Data analyses explored the occurrence of inpatient mortality, employing general linear mixed models to identify the effects of patients', health care processes', healthcare units' and municipalities' characteristics on it.

**Results:**

About 70% of the COVID-19 hospitalizations in Brazil were covered by the SUS, which attended the more vulnerable population groups and had worse inpatient mortality. In general, non-SUS private and philanthropic hospitals, mostly reimbursed by healthcare insurance plans accessible for more privileged socioeconomic classes, presented the best outcomes. Southern Brazil had the best performance among the macro-regions. Black and indigenous individuals, residents of lower HDI municipalities, and those hospitalized out of their residence city presented higher odds of inpatient mortality. Moreover, adjusted inpatient mortality rates were higher in the pandemic peak moments and were significantly reduced after COVID-19 vaccination reaching a reasonable coverage, from July 2021.

**Conclusions:**

COVID-19 exposed socioeconomic and healthcare inequalities and the importance and weaknesses of SUS in Brazil. This work indicates the need to revert the disinvestment in the universal public system, a fundamental policy for reduction of inequities in the country.

**Supplementary Information:**

The online version contains supplementary material available at 10.1186/s12939-023-02037-8.

## Introduction

Although inequalities in health and healthcare have been documented for decades, the COVID-19 pandemic stressed the social gradient from exposure to ability to treat infection and to avoid deaths, with the worst outcomes occurring in vulnerable population groups, even in high income countries [1–3]. Mortality due to COVID-19 has been shown to be unequally distributed by race, ethnicity, socioeconomic status, and degree of access to adequate health services [4–8], and, under these conditions, universal health systems could be expected to play a protective role in tackling healthcare access barriers, avoiding undesirable outcomes [9, 10].

Globally, healthcare systems had to face numerous challenges in dealing with COVID-19 pandemic, which highlighted their strengths and weaknesses in relation to factors such as financing, coordination capacity, infrastructure, healthcare delivery, and health workforce [9, 11, 12]. The pandemic found Brazil in a context of political polarizations and the universal and public Unified Health System (SUS) with problems accumulated over time, due, among other reasons, to low investments, and disparities in resource distribution. Furthermore, since 2014, the Brazilian economic retraction had imposed a throwback in the improvement of social conditions observed in the previous years and relevant constraints to SUS performance [13, 14].

Despite being universal, public and tasked with executing numerous public health interventions, the SUS, in practice, shares the healthcare delivery function with a private sector that is mostly remunerated through private healthcare insurance companies [15], in general accessible to more privileged socioeconomic groups. About a quarter of the Brazilian population has a private health insurance, there existing more concentration of health insurance beneficiaries in the wealthiest regions (South and Southeast), where the private healthcare sector is more expanded.

The preparedness and response of the healthcare system as a whole to the pandemic scenario was strongly affected by the large socioeconomic inequities across the country [12, 16]. Financial resources and strategies to expand physical infrastructure and workforce were implemented, but lacking federal government coordination and governance incurred numerous undesirable consequences [16]. Inpatient healthcare, critical during the pandemic, was delivered by a network of public, private, and philanthropic (private nonprofit) providers that had to adjust themselves to the demands presented.

The SUS network involves an expressive number of federal, state, and municipal public hospitals, but it is partially dependent on private and philanthropic providers, which, in turn, may provide services to SUS or strictly provide services in the private sector. The philanthropic hospitals, specifically, which may be or not part of conglomerates, are mostly required to provide healthcare services to SUS, and, majorly, are essential inpatient care providers to the public system across the country [17]. However, other arrangements, involving the segmentation of conglomerates or the provision of other services, have allowed part of them, broadly recognized for high healthcare quality standards, to accomplish the requirements for maintenance of the philanthropy certificate without directly providing healthcare to SUS patients. It is still noteworthy that there is a small contingent of public hospitals, related to military and civil government employee organizations, which are not SUS providers.

The Brazilian hospital network is extensive, but the geographic distribution is unequal, rarer in the interior of the country than in the capitals, in rural areas than in urban ones, in poorer states than in richer ones. Such providers were not fully able to overcome the inherent limitations stemming from the prevalence of small, low-complexity units, while more complex units were concentrated in some metropolitan areas. Given the existing significant disparities in supply and access between SUS and non-SUS users, as well as among different geographic areas [18], the pandemic further intensified the detrimental impact of these profound inequities and imbalances on the geographical and social allocation of healthcare resources [18–20]. These inequities certainly were expressed in the use variation and outcomes of the healthcare among SUS and non-SUS users, such as hospital mortality.

Given the complexities around COVID-19 and the challenges it brought to the healthcare system, there is keen interest in understanding the differences in the management and outcomes of COVID-19 patients treated at the individual and hospital levels of different structures [21–25]. Studies have shown variation in the adjusted COVID-19 inpatient mortality by age, sex, race, clinical condition, country areas and hospitals [26–30]. In Brazil, results from studies based exclusively on data from public hospitals [18] or specific private hospitals [31] raise questions about the likelihood of relevant difference in COVID-19 inpatient mortality between hospitals in the public and private sector. In fact, there is great heterogeneity within and between public and private systems and their hospital networks, resulting in variation in healthcare quality provided to SUS and private health insurance users. There is also some evidence that hospitals performed better in contexts in which the prevalence of COVID-19 was lower [19, 25]. Furthermore, vaccination coverage and learning on how to care for COVID-19 cases impacted, over time, the occurrence of hospitalizations, the profile of patients hospitalized and healthcare outcomes [23, 30, 32–36].

In Brazil, a relevant aspect not yet sufficiently explored was the variation in COVID-19 inpatient mortality across categories of hospitals combining ownership and participation in the SUS as an inpatient care provider. Thus, this work was aimed at offering an overview of COVID-19 inpatient mortality during the pandemic in Brazil, exploring factors associated with its variations and, specifically, differences across public, private (for-profit) and philanthropic (private non-profit) inpatient healthcare units, providers, and non-providers of services to the SUS.

## Methods

### Study design

This is a cross-sectional study based on Brazilian publicly available secondary data (Supplement 1 – STROBE checklist) [37].

### Setting and participants

It comprises COVID-19 hospitalizations registered in Brazil from February 2020 to December 2022 for 18-year-old patients and older in inpatient healthcare units with at least 100 COVID-19 hospitalizations in the period.

### Data sources

The study was based on secondary data of the Brazilian Ministry of Health and the Brazilian Institute of Geography and Statistics (IBGE). The main data source was the SIVEP-Gripe, which comprises prospectively collected data on severe acute respiratory illness records. The SIVEP-Gripe data were accessed on the website https://opendatasus.saude.gov.br. The data for 2020 were downloaded on May 16, 2022, while the data for 2021 and 2022 were downloaded on January 24, 2023. Observations from the SIVEP-Gripe database were filtered on variables indicating hospitalization occurrence and final case classification as 'SRAG by COVID-19'. Additionally, only registers for individuals who were at least 18 years old were kept, and registers with an unknown outcome were excluded.

The study also utilized data from the National Record of Health Establishments (CNES) to gather comprehensive information about the healthcare units, from the SUS Hospitalization Information System (SIH-SUS) to identify hospitals that had COVID-19 hospitalizations covered by the SUS, and data about Brazilian municipalities came from IBGE. The first two are available on the DATASUS site https://datasus.saude.gov.br/transferencia-de-arquivos/). The last are available on the site https://cidades.ibge.gov.br.

### Main outcome

The dependent variable was COVID-19 inpatient mortality, defined based on the discharge outcome registered in SIVEP-Gripe. The event of interest was the occurrence of death due to COVID-19.

### Independent variables

The main independent variable in the study, “inpatient healthcare unit category”, was defined by combining the inpatient care unit ownership and information on COVID-19 inpatient care provision to SUS, classifying hospitals into six groups: public SUS, public non-SUS, private SUS, private non-SUS, philanthropic SUS and philanthropic non-SUS.

Accounting for the notification unit code available in SIVEP-Gripe, we associated the establishment type, number of beds and ownership of the healthcare unit available in CNES. Data not available in the CNES files were input based on information from the site https://cnes.datasus.gov.br. We kept only observations in SIVEP-Gripe for inpatient care units belonging to the following categories: general and specialized hospitals, mixed units (outpatient units with beds for inpatient care) and general and specialized emergency centers.

Considering the variation in the inpatient health care unit’s number of beds over time, we assumed the highest number in the study period, believing that categorization would attenuate discrepancies. We opted to identify inpatient care units providing care to the SUS considering those with COVID-19 hospitalizations registered in the Hospital Information system (SIH) of the SUS in the study period.

Variations in inpatient mortality, first, are explained by differences in the cases’ severity. In this sense, we also considered the effects of age, sex and total number and presence of specific comorbidities – traditional case-mix variables –, adding to them other patient socioeconomic variables (race/color, education) and healthcare process variables (length of stay, ICU use, ventilatory support use) able to affect the outcome reflecting social and healthcare access and effectiveness inequalities or severity not captured by the case-mix ones. All these variables were obtained from SIVEP-Gripe, from which we also accounted for the week of the date of the first symptoms, the symptoms, the notification unit city, and the patient residence city. Temporal occurrence of the COVID-19 event was classified based on the first symptom date. We accounted for the pandemic phases proposed in a balance made in February 2022 [38]. From IBGE, we obtained the population and the Human Development Index of Brazilian municipalities (HDI).

### Data analyses

Data management and analyses were performed using SAS^®^ statistical package, version 9.4.

We explored the occurrence of hospitalizations and inpatient mortality over time and across hospital groups defined by ownership and participation in SUS, grouped as inpatient care unit categories in the country and macro-regions. We also explored the sociodemographic profiles of the patients assisted in the different inpatient care unit groups.

Bivariate analyses were performed to describe the occurrence of COVID-19 inpatient mortality across variables reflecting patients', health care processes', healthcare units' and municipalities' characteristics. In the multivariate analyses, we employed general linear mixed models (GLIMMIX) to identify the independent effects of these variables, at different levels, on COVID-19 inpatient mortality. We run models for Brazil and its macro-regions, keeping comparable final models with relevant variables/categories.

## Results

SIVEP-Gripe datasets obtained for 2020, 2021 and 2022 included 1,200,981, 1,733,594 and 555,793 observations, respectively. We kept a set of 1,885,161 observations that adhered to four following criteria: occurrence (first symptoms) from February 2020; confirmed COVID-19 case; hospitalization; patient at least 18 years old; and known discharge outcome. After merging SIVEP-Gripe with CNES, we excluded those observations for which the notification unit codes available in SIVEP-Gripe neither were identified nor corresponded to an inpatient care unit (general hospital, specialized hospital, mixed unit, general emergency center and specialized emergency center), as well as those for which the inpatient care unit had fewer than 100 hospitalizations in the period. We also excluded observations in which the notification unit city code or the patient residence city code were unknown. The final dataset had 1,615,428 observations, corresponding to approximately 86% of registers selected in applying the initial selection criteria.

**Fig. 1:**
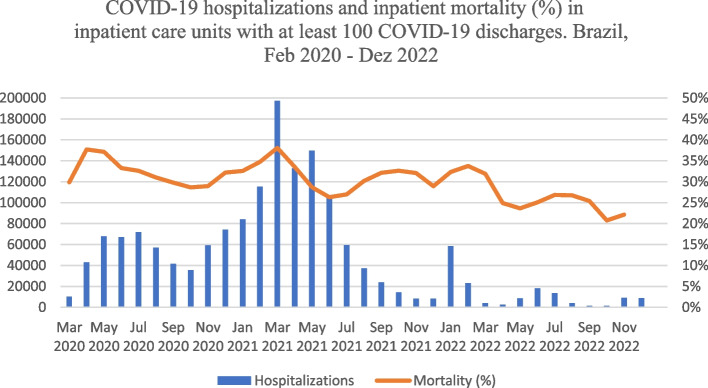
COVID-19 hospitalizations and inpatient mortality (%) in inpatient care units with at least 100 COVID-19 discharges. Brazil, Feb 2020 - Dez 2022. Source: SIVEP Gripe - Sistema de Informação de Vigilância Epidemiológica da Gripe

Figure 1 provides an overview of COVID-19 hospitalizations and inpatient mortality in the period between February 2020 and December 2022. We underline the observation of three waves: the first, from February to August 2020, in which the pandemic was initially concentrated in a few metropoles and later expanded; the second, from December 2020 to June 2021, which was broadly spread throughout the country, reaching the highest numbers of cases, hospitalizations and deaths; and the third, in the beginning of 2022. From the middle of 2021, and, especially, after the third wave, a vertiginous reduction in the number of hospitalizations was evident and somewhat in inpatient mortality.

Table 1 presents the distribution of COVID-19 hospitalizations and inpatient mortality across the categories of inpatient care units defined in the SUS in Brazil and in the macro- regions. It is reasonable to say, considering the universe of discharges analyzed, that SUS coverage surpassed 70% in the country, with regional variations.

**Table 1:** COVID-19 adult hospitalizations and inpatient mortality per inpatient care unit categories. Brazil and macro-regions of the country, February 2020 – December 2022

Region/ inpatient care unit category	Units	Discharges		Inpatient mortality (%)		
		N	%	Proportion	STD	95% CI
Brazil						
Total	2,290	1,615,428	100.0	32.1	46.7	32.0; 32.1
Public SUS	876	632,024	39.1	37.5	48.4	37.3; 37.6
Public non-SUS	23	13,599	0.8	32.2	46.7	31.4; 33.0
Private SUS	106	70,175	4.3	32.0	46.6	31.6; 32.3
Private non-SUS	462	346,035	21.4	24.6	43.1	24.4; 24.7
Philanthropic SUS	738	475,456	29.4	32.1	46.7	32.0; 32.3
Philanthropic non-SUS	85	78,139	4.8	21.3	41.0	21.1; 21.6
North						
Total	189	99,365	100.0	36.1	48.0	35.8; 36.4
Public SUS	138	78,388	78.9	35.4	47.8	35.1; 35.8
Public non-SUS	1	508	0.5	24.6	43.1	20.8; 28.4
Private SUS	11	3,151	3.2	39.7	48.9	38.0; 41.4
Private non-SUS	21	8,447	8.5	45.9	49.8	44.8; 46.9
Philanthropic SUS	11	4,735	4.8	33.7	47.3	32.4; 35.1
Philanthropic non-SUS	7	4,136	4.2	30.1	45.9	28.7; 31.5
Northeast						
Total	427	247,242	100.0	36.7	48.2	36.4; 36.8
Public SUS	250	148,839	60.2	40.6	49.1	40.3; 40.8
Public non-SUS	5	1,058	0.4	35.0	47.7	32.1; 37.8
Private SUS	32	12,761	5.2	36.6	48.2	35.7; 37.4
Private non-SUS	72	50,965	20.6	27.0	44.4	26.6; 27.4
Philanthropic SUS	63	29,981	12.1	33.7	47.3	33.1; 34.2
Philanthropic non-SUS	5	3,638	1.5	32.9	47.0	31.4; 34.4
Southeast						
Total	1056	803,598	100.0	31.6	46.5	31.5; 31.7
Public SUS	329	253,990	31.6	39.9	49.0	39.7; 40.1
Public non-SUS	12	9,613	1.2	34.2	47.4	33.3; 35.2
Private SUS	18	13,711	1.7	28.5	45.1	27.7; 29.2
Private non-SUS	270	217,564	27.1	24.2	42.8	24.0; 24.4
Philanthropic SUS	374	256,557	31.9	32.3	46.8	32.1; 32.5
Philanthropic non-SUS	53	52,163	6.5	19.2	39.4	18.8; 19.5
South						
Total	398	301,996	100.0	30.0	45.8	29.8; 30.1
Public SUS	67	68,235	22.6	31.6	46.5	31.2; 31.9
Public non-SUS	3	928	0.3	30.5	46.1	27.5; 33.5
Private SUS	15	18,445	6.1	34.4	47.5	33.7; 35.0
Private non-SUS	46	34,069	11.3	20.0	40.0	19.6; 20.5
Philanthropic SUS	255	165,912	54.9	31.4	46.4	31.2; 31.6
Philanthropic non-SUS	12	14,407	4.8	23.9	42.7	23.2; 24.6
Midwest						
Total	220	163,227	100.0	29.0	45.3	28.7; 29.2
Public SUS	92	82,572	50.6	31.2	46.3	30.9; 31.5
Public non-SUS	2	1,492	0.9	20.9	40.7	18.8; 23.0
Private SUS	30	22,107	13.5	28.4	45.1	27.8; 29.0
Private non-SUS	53	34,990	21.4	22.8	41.9	22.3; 23.2
Philanthropic SUS	35	18,271	11.2	33.9	47.3	33.2; 34.6
Philanthropic non-SUS	8	3,795	2.3	20.9	40.7	19.6; 22.2

Source: SIVEP Gripe - Sistema de Informação de Vigilância Epidemiológica da Gripe

The study excluded inpatient care units with less than 100 COVID-19 hospitalizations in the period

Southeastern Brazil was the region in which the participation of non-SUS private and philanthropic hospitals was the highest, corresponding to 270 hospitals and 27.1% of the COVID-19 hospitalizations and 53 hospitals and 6.5% of the COVID-19 hospitalizations, respectively. The SUS public hospitals were responsible for most COVID-19 hospitalizations in the North (78.9%) and in the Northeast (60.2%) and about half in the Midwest (50.6%), while the SUS philanthropic hospitals were responsible for the majority of COVID-19 hospitalizations in the South (54.9%) and for the largest volume (31.9%) in the Southeast. The non-SUS public hospitals are mostly military and comprised relatively small numbers of hospitalizations. Additionally, it is worth noting the diverse mix of hospitals within the private SUS group, ranging from those with a limited number of beds available for SUS patients to those that encompass the totality. In the group, there are also 15 public teaching hospitals, among them 13 administered by the *Empresa Brasileira de Serviços Hospitalares* (EBSERH) [Brazilian Company of Hospital Services], a kind of management arrangement [39].

Regarding hospital performance measured in terms of crude COVID-19 inpatient mortality, the non-SUS philanthropic and private hospitals presented the best results in the country, which was approximately reproduced in the macro-regions, except for the North. Grossly, the worst crude inpatient mortality rates (%) were observed for the SUS public hospitals in Brazil (37.5; 95%CI 37.3; 37.6), in the Northeast (40.6; 95%CI 40.3; 40.8) and in the Southeast (39.9; 95%CI 39.7; 40.1); for the non-SUS private hospitals in the North (45.9; 95%CI 44.8; 46.9); for the SUS private hospitals in the South (34.4; 95%CI 33.7; 35.0); and for the SUS philanthropic hospitals in the Midwest (33.9; 95%CI 33.2; 34.6). Although a more specific analysis of different government sphere public units was not our focus, we found in some explorations that they were predominantly municipal (55.4%), and state (38.5%) SUS providers, which comprised, respectively, 46.1% and 48.4% of the COVID-19 discharges in the public sector (Supplement 2). Overall, in the country, we did not identify significant crude inpatient mortality differences among the different government level SUS providers (federal – 38.2; 95%CI 37.5; 38.9; state – 37.6; 95%CI 37.4; 37.8; and municipal – 37.3, 95%CI 37.1; 37.4).

Despite the elevated frequency of missing data for race/color and education level, Table 2 suggests a higher concentration of blacks, indigenous, and lower-educated patients in the SUS public hospitals, while the proportions of whites were higher in the non-SUS philanthropic hospitals and in the SUS philanthropic hospitals, in this case possibly reflecting the high participation of these in Southern Brazil. The patients with tertiary education were more frequent in the non-SUS private and philanthropic hospitals. The non-SUS public hospitals, mostly military, presented higher proportions of males and older patients.

**Table 2:** Distribution of COVID-19 hospitalizations across sociodemographic variables per inpatient care unit types. Brazil, February 2020 – December 2022

Variable	Public SUS (*N=*632,024)		Public non-SUS (*N=*13,599)		Private SUS (*N=*70,175)		Private non-SUS (*N=*346,035)		Philanthropic SUS (*N=*475,456)		Philanthropic non-SUS (*N=*78,139)		Total (*N=*1,615,428)	
	*N*	%	*N*	%	*N*	%	*N*	%	*N*	%	*N*	%	*N*	%
Age (years)														
18-39	91,299	14.5	954	7.0	10,543	15.0	56,805	16.4	65,340	13.7	11,304	14.5	236,245	14.6
40-49	95,406	15.1	1,723	12.7	11,140	15.9	60,794	17.6	70,354	14.8	13,137	16.8	252,554	15.6
50-59	126,816	20.1	2,627	19.3	13,829	19.7	65,028	18.8	95,250	20.0	15,143	19.4	318,693	19.7
60-69	132,293	20.9	2,485	18.3	13,901	19.8	59,263	17.1	97,868	20.6	13,864	17.7	319,674	19.8
70-79	107,563	17.0	2,984	21.9	11,696	16.7	51,763	15.0	81,569	17.2	12,187	15.6	267,762	16.6
≥ 80	78,647	12.4	2,826	20.8	9,066	12.9	52,382	15.1	65,075	13.7	12,504	16.0	220,500	13.7
Sex														
Female	284,233	45.0	5,558	40.9	30,948	44.1	149,920	43.3	215,948	45.4	32,895	42.1	719,502	44.5
Male	347,707	55.0	8,039	59.1	39,222	55.9	196,102	56.7	259,480	54.6	45,239	57.9	895,789	55.5
Unknown	84	0.0	2	0.0	5	0.0	13	0.0	28	0.0	5	0.0	137	0.0
Race/color														
White	184,984	29.3	4,318	31.8	29,544	42.1	149,990	43.4	305,233	64.2	39,738	50.9	713,807	44.2
Black	30,895	4.9	764	5.6	2,836	4.0	8,431	2.4	19,975	4.2	1,796	2.3	64,697	4.0
Asian	6,969	1.1	102	0.7	687	1.0	3,538	1.0	3,321	0.7	750	1.0	15,367	1.0
Mixed race	306,459	48.5	3,073	20.6	22,200	31.6	71,900	20.8	103,456	21.8	11,698	15.0	518,786	32.1
Indigenous	1,624	0.3	20	0.2	98	0.1	206	0.1	545	0.1	35	0.0	2,528	0.2
Unknown	101,093	16.0	5,322	39.1	14,810	21.1	111,970	32.4	42,926	9.0	24,122	30.9	300,243	18.6
Education														
No schooling	19,780	3.1	55	0.4	1,461	2.1	1,689	0.5	11,152	2.4	302	0.4	34,439	2.1
Primary	65,074	10.3	567	4.2	4,889	7.0	13,389	3.9	66,513	14.0	2,702	3.5	153,134	9.5
Lower secondary	44,230	7.0	571	4.2	6,221	8.9	12,684	3.7	43,479	9.1	2,355	3.0	109,540	6.8
Upper secondary	63,612	10.1	3,077	22.6	8,535	12.2	44,049	12.7	63,328	13.3	7,614	9.7	190,215	11.8
Tertiary	17,216	2.7	835	6.1	4,914	7.0	35,706	10.3	24,750	5.2	9,152	11.7	92,573	5.7
Unknown	422,112	66.8	8,494	62.5	44,155	62.9	238,518	68.9	266,234	56.0	56,014	71.7	1,035,527	64.1

Source: SIVEP Gripe - Sistema de Informação de Vigilância Epidemiológica da Gripe

The study excluded inpatient care units with less than 100 COVID-19 hospitalizations in the period

Table 3 shows the results of the bivariate analyses describing the relationship between inpatient mortality and various potential explanatory variables for the country. In general terms, we highlight the socioeconomic and severity risk variation associated with age increase, the presence of comorbidities, race/color, use of ICU and use of ventilatory support, considering that the last two are healthcare process variables. Data on education suggest a decreasing gradient of inpatient mortality as education increases, but the appreciation is compromised by the high level of missing information. Differentiation of the inpatient mortality risk based on the number of symptoms was not identified. A very high occurrence of deaths was observed among those who entered and left the inpatient care units on the same day. Moreover, the results suggest lower mortality among patients living in cities with higher HDI.

**Table 3:** Bivariate analyses of inpatient mortality with patient-, inpatient care unit-, and municipality-level variables

Variable	*N*	%	Dead		Alive	
			*N*	%	*N*	%
Inpatient care unit category						
Public SUS	632,024	39.1	236,743	37.5	395,281	62,5
Public non-SUS	13,599	0.8	4,379	32.2	9,220	67,8
Private SUS	70,175	4.3	22,431	32.0	47,744	68,0
Private non-SUS	346,035	21.4	85,092	24.6	260,943	75,4
Philanthropic SUS	475,456	29.4	152,856	32.1	322,600	67,9
Philanthropic non-SUS	78,139	4.8	16,679	21.3	61,460	78,7
Age (years)						
18-39	236,245	14.6	29,899	12.7	206,346	87,3
40-49	252,554	15.6	46,308	18.3	206,246	81,7
50-59	318,693	19.7	82,185	25.8	236,508	74,2
60-69	319,674	19.8	118,350	37.0	201,324	63,0
70-79	267,762	16.6	122,926	45.9	144,836	54,1
≥ 80	220,500	13.6	118,512	53.7	101,988	46,3
Sex						
Female	719,502	44.5	227,286	31.6	492,216	68,4
Male	895,789	55.5	290,842	32.5	604,947	67,5
Unknown	137	0.0	52	38.0	85	62,0
Race/color						
White	713,807	44.2	230,070	32.2	483,737	67,8
Black	64,697	4.0	24,495	37.9	40,202	62,1
Asian	15,367	1.0	4,850	31.6	10,517	68,4
Mixed race	518,786	32.1	176,611	34.0	342,175	66,0
Indigenous	2,528	0.2	878	34.7	1,650	65,3
Unknown	300,243	18.6	81,276	27.1	218,967	72,9
Education						
No schooling	34,439	2.1	17,405	50.5	17,034	49,5
Primary	153,134	9.5	66,065	43.1	87,069	56,9
Lower secondary	109,540	6.8	40,228	36.7	69,312	63,3
Upper secondary	190,215	11.8	53,020	27.9	137,195	72,1
Tertiary	92,573	5.7	22,318	24.1	70,255	75,9
Unknown	1,035,527	64.1	319,144	30.8	716,383	69,2
COVID-19 symptoms						
< 5	917,282	56.8	295,066	32.2	622,216	67,8
≥ 5	698,146	43.2	223,114	32.0	475,032	68,0
Comorbidity – obesity						
Yes	135,333	8.4	51,105	37.8	84,228	62,2
No	1,480,095	91.6	467,075	31.6	1,013,020	68,4
Comorbidity – cardiopathy						
Yes	530,766	32.9	216,064	40.7	314,702	59,3
No	1,084,662	67.1	302,116	27.9	782,546	72,1
Comorbidity – diabetes						
Yes	372,910	23.1	154,371	41.4	218,539	58,6
No	1,242,518	76.9	363,809	29.3	878,709	70,7
Comorbidity – hematologic disease						
Yes	10,842	0.7	4,817	44.4	6,025	55,6
No	1,604,586	99.3	513,363	32.0	1,091,223	68,0
Comorbidity – Down syndrome						
Yes	3,928	0.2	1,613	41.1	2,315	58,9
No	1,611,500	99.8	516,567	32.1	1,094,933	67,9
Comorbidity – hepatic disease						
Yes	13,017	0.8	6,639	51.0	6,378	49,0
No	1,602,411	99.2	511,541	31.9	1,090,870	68,1
Comorbidity - asthma						
Yes	38,953	2.4	11,092	28.5	27,861	71,5
No	1,576,475	97.6	507,088	32.2	1,069,387	67,8
Comorbidity – pneumopathy						
Yes	54,712	3.4	27,368	50.0	27,344	50,0
No	1,560,716	96.6	490,812	31.4	1,069,904	68,6
Comorbidity – neurologic disease						
Yes	58,806	3.6	30,108	51.2	28,698	48,8
No	1,556,622	96.4	488,072	31.4	1,068,550	68,6
Comorbidity – kidney disease						
Yes	59,105	3.7	31,718	53.7	27,387	46,3
No	1,556,323	96.3	486,462	31.3	1,069,861	68,7
Comorbidity – immunodepression						
Yes	39,630	2.5	18,366	46.3	21,264	53,7
No	1,575,798	97.5	499,814	31.7	1,075,984	68,3
Comorbidity – other disease						
Yes	452,071	28.0	180,210	39.9	271,861	60,1
No	1,163,357	72.0	337,970	29.1	825,387	70,9
Number of comorbidities						
0	602,521	37.3	130,978	21.7	471,543	78,3
1	487,026	30.1	157,975	32.4	329,051	67,6
2	347,052	21.5	140,916	40.6	206,136	59,4
3	137,124	8.5	65,462	47.7	71,662	52,3
≥ 4	41,705	2.6	22,849	54.8	18,856	45,2
ICU use						
Yes	580,789	36.0	323,627	55.7	257,162	44,3
No	1,034,639	64.0	194,553	18.8	840,086	81,2
Ventilatory support use						
Yes, invasive	304,150	18.8	232,945	76.6	71,205	23,4
Yes, non-invasive	832,351	51.5	190,548	22.9	641,803	77,1
No	288,385	17.9	33,824	11.7	254,561	88,3
Unknown	190,542	11.8	60,863	31.9	129,679	68,1
Length of stay (days)						
0	32,688	2.0	19,847	60.7	12,841	39,3
≥1	1,519,154	94.0	489,477	32.2	1,029,677	67.8
Unknown	63,586	3.9	8,856	13.9	54,730	86,1
Patient residence city’s HDI						
Very low (<0.500)	1,232	0.1	327	26.5	905	73,5
Low (0.500-0.599)	54,924	3.4	20,938	38.1	33,986	61,9
Medium (0.600-0.699)	196,998	12.2	71,437	36.3	125,561	63,7
High (0.700-0.799)	969,716	60.0	317,107	32.7	652,609	67,3
Very high (≥0.800)	392,558	24.3	108,371	27.6	284,187	72,4
Inpatient care unit’s COVID-19 hospitalizations 2020-2022						
100-299	159,081	9.8	57,993	36.5	101,088	63,5
300-599	237,066	14.7	81,593	34.4	155,473	65,6
600-999	287,125	17.8	93,523	32.6	193,602	67,4
1000-3999	762,847	47.2	230,365	30.2	532,482	69,8
≥ 4000	169,309	10.5	54,706	32.3	114,603	67,7
Hospital beds						
< 30	68,187	4.2	18,704	27.4	49,483	72,6
30-49	107,077	6.6	29,718	27.8	77,359	72,2
50-99	290,920	18.0	84,583	29.1	206,337	70,9
100-199	535,855	33.2	172,358	32.2	363,497	67,8
200-399	389,708	24.1	132,935	34.1	256,773	65,9
≥ 400	210,175	13.0	74,642	35.5	135,533	64,5
Unknown	13,506	0.8	5,240	38.8	8,266	61,2
Healthcare unit type						
General hospital	1,521,877	94.2	484,267	31.8	1,037,610	68,2
Specialized hospital	49,615	3.1	17,772	35.8	31,843	64,2
Mixed unit	9,880	0.6	2,810	28.4	7,070	71,6
General emergency center	25,775	1.6	10,258	39.8	15,517	60,2
Specialized emergency center	8,281	0.5	3,073	37.1	5,208	62,9
Country macro-region						
North	99,365	6.2	35,872	36.1	63,493	63,9
Northeast	247,242	15.3	90,521	36.6	156,721	63,4
Southeast	803,598	49.7	253,971	31.6	549,627	68,4
South	301,996	18.7	90,536	30.0	211,460	70,0
Midwest	163,227	10.1	47,280	29.0	115,947	71,0
Inpatient care’s city						
Same of patient’s residence	1,146,988	71.0	355,753	31.0	791,235	69,0
In patients’ residence Health Region	250,997	15.5	87,259	34.8	163,738	65,2
Another Health Region	217,443	13.5	75,168	34.6	142,275	65,4
Inpatient care unit’s city size						
<20000 inhabitants	32,636	2.0	6,601	20.2	26,035	79,8
20000-49999 inhabitants	122,254	7.6	34,725	28.4	87,529	71,6
50000-99999 inhabitants	166,215	10.3	53,954	32.5	112,261	67,5
100000-999999 inhabitants	696,049	43.1	232,920	33.5	463,129	66,5
≥ 1000000 inhabitants	598,274	37.0	189,980	31.8	408,294	68,2
Pandemic period						
Feb 2020 – Mai 2020 (wave 1.1)	121,022	7.5	44,440	36.7	76,582	63.3
Jun 2020 – Ago 2020 (wave 1.2 - expansion)	196,019	12.1	63,447	32.4	132572	67.6
Sep 2020 – Nov 2020	136,103	8.4	396,24	29.1	96,479	70,9
Dec 2020 – Fev 2021 (wave 2.1)	273,395	16.9	912,73	33.4	182,122	66,6
Mar 2021 – Apr 2021 (wave 2.2)	329,850	20.4	119,572	36.3	210,278	63,7
May 2021 – Jun 2021 (wave 2.3)	254,463	15.8	70,480	27.7	183,983	72,3
Jul 2021 – Dec 2021	151,421	9.4	44,646	29.5	106,775	70,5
Jan 2022 – Feb 2022 (wave 3)	81,595	5.1	26,706	32.7	54,889	67,3
Mar 2022 – Dec 2022	71,560	4.4	17,992	25.1	53,568	74,9

Source: SIVEP Gripe - Sistema de Informação de Vigilância Epidemiológica da Gripe

The study excluded inpatient care units with less than 100 COVID-19 hospitalizations in the period

By examining variables at the hospital level, non-adjusted results suggest an increase in inpatient mortality as the number of hospital beds increases, emphasizing the notably high mortality observed in a particular private hospital where the notified number of beds in CNES was evidently inconsistent with the high volume of COVID-19 hospitalizations reported, which is why we considered the number of beds as unknown. Regarding inpatient care unit types, unadjusted mortality seemed higher in emergency centers and lower in mixed units.

COVID-19 inpatient mortality was also shown to be positively related to hospitalization outside of the patient’s city of residence and the size of the hospital's municipality. The unadjusted results indicate higher mortalities in the North and the Northeast and lower mortalities in the South and Midwest compared to the Southeast. In relation to the pandemic period, the waves tend to present higher inpatient mortalities.

Table 4 shows the odds ratios and corresponding 95% confidence intervals for the explanatory variables included in the final GLIMMIX models for Brazil and the five macro-regions. Among the intermediary explorations, we assessed the inclusion of the variables “race/color” and “education”, with high prevalence of missing values not equally distributed among the inpatient care unit groups, and the role of healthcare process variables on the results (Supplement 3).

**Table 4:** Generalized linear mixed models: factors associated with COVID-19 inpatient mortality in Brazil and in the country’s macro-regions. Brazil, Feb 2020 – Dec 2022

Variable	Brazil		North		Northeast		Southeast		South		Midwest	
	OR	95%CI	OR	95%CI	OR	95%CI	OR	95%CI	OR	95%CI	OR	95%CI
Inpatient healthcare unit category (ref: Public SUS)												
Public non-SUS	0.58	0.46; 0.74	0.38	0.13; 1,12	0.35	0.18; 0.67	0.61	0.44; 0.84	0.65	0.34; 1.23	0.98	0.53; 1.83
Private SUS	0.57	0.47; 0.68	0.86	0.48; 1.54	0.51	0.34; 0.76	0.45	0.29; 0.68	1.00	0.66; 1.51	0.55	0.39; 0.79
Private non-SUS	0.43	0.39; 0.48	0.59	0.37; 0.94	0.43	0.32; 0.57	0.37	0.32; 0.44	0.60	0.45; 0.80	0.52	0.38; 0.70
Philanthropic SUS	0.89	0.80; 0.98	0.69	0.39; 1.23	0.95	0.71; 1.28	0.73	0.63; 0.85	1.19	0.97; 1.47	1.05	0.76; 1.46
Philanthropic non-SUS	0.38	0.31; 0.46	0.44	0.22; 0.90	0.98	0.38; 2.50	0.34	0.26; 0.44	0.38	0.24; 0.59	0.33	0.18; 0.61
Age (ref: 18-39 years)												
40-49 years	1.45	1.42; 1.48	1.65	1.54; 1.77	1.42	1.35; 1.49	1.42	1.38; 1.46	1.48	1.41; 1.55	1.48	1.39; 1.57
50-59 years	2.09	2.05; 2.13	2.53	2.36; 2.71	1.99	1.91; 2.08	2.06	2.01; 2.12	2.18	2.09; 2.28	2.02	1.91; 2.13
60-69 years	3.45	3.39; 3.52	4.41	4.12; 4.71	3.14	3.00; 3.28	3.42	3.33; 3.51	3.63	3.48; 3.80	3.33	3.15; 3.52
70-79 years	5.64	5.53; 5.74	7.09	6.62; 7.60	4.93	4.71; 5.15	5.52	5.37; 5.66	6.62	6.32; 6.93	5.14	4.85; 5.45
≥ 80 years	10.95	10.74; 11.17	10.96	10.18; 11.80	8.79	8.39; 9.20	10.80	10.51; 11.10	15.19	14.46; 15.94	9.62	9.04; 10.24
Male (yes vs. no)	1.21	1.20; 1.22	1.16	1.12; 1.20	1.14	1.11; 1.16	1.23	1.21; 1.24	1.22	1.20; 1.25	1.23	1.20; 1.27
Race/color (ref: mixed race/white/ Asian/unknown)												
Black	1.19	1.16; 1.21	1.09	0.98; 1.22	1.26	1.18; 1.33	1.22	1.18; 1.25	1.09	1.02; 1.16	1.12	1.03; 1.21
Indigenous	1.18	1.06; 1.31	1.65	1.39; 1.96	0.96	0.71; 1.29	0.67	0.51; 0.87	1.16	0.84; 1.61	1.26	0.97; 1.65
Comorbidities (ref: 0)												
1	1.30	1.28; 1.31	1.19	1.14; 1.24	1.27	1.24; 1.31	1.26	1.24; 1.28	1.56	1.51; 1.61	1.34	1.29; 1.40
2	1.49	1.47; 1.51	1.26	1.20; 1.33	1.37	1.32; 1.41	1.46	1.43; 1.48	1.85	1.79; 1.91	1.61	1.54; 1.68
≥ 3	1.69	1.66; 1.72	1.28	1.17; 1.40	1.41	1.35; 1.48	1.67	1.63; 1.71	2.22	2.13; 2.32	1.86	1.75; 1.98
Down syndrome (yes vs. no)	1.18	1.09; 1.28	1.30	0.95; 1.76	1.23	1.00; 1.50	1.29	1.15; 1.45	1.11	0.92; 1.34	0.79	0.59; 1.07
Obesity (yes vs. no)	1.18	1.16; 1.20	1.55	1.42; 1.69	1.10	1.05; 1.15	1.24	1.21; 1.27	1.04	1.00; 1.08	1.27	1.20; 1.34
Hematologic disease (yes vs. no)	1.26	1.19; 1.32	1.04	0.80; 1.35	1.23	1.07; 1.42	1.28	1.20; 1.37	1.32	1.18; 1.48	1.09	0.91; 1.31
Hepatic disease (yes vs. no)	1.66	1.59; 1.74	1.30	1.05; 1.62	1.86	1.66; 2.08	1.69	1.58; 1.80	1.67	1.51; 1.84	1.50	1.28; 1.75
Neurologic disease (yes vs. no)	1.62	1.59; 1.66	1.20	1.06; 1.37	1.41	1.32; 1.50	1.58	1.54; 1.63	1.91	1.83; 2.00	1.35	1.24; 1.46
Pneumopathy (yes vs. no)	1.25	1.23; 1.28	1.32	1.16; 1.51	1.13	1.05; 1.21	1.25	1.22; 1.29	1.25	1.19; 1.30	1.21	1.11; 1.31
Kidney disease (yes vs. no)	1.55	1.52; 1.59	1.52	1.37; 1.69	1.59	1.50; 1.68	1.56	1.51; 1.61	1.51	1.43; 1.59	1.56	1.45; 1.69
Immunodepression (yes vs. no)	1.98	1.93; 2.03	1.89	1.65; 2.15	1.93	1.80; 2.08	1.92	1.85; 2.00	2.25	2.12; 2.39	1.80	1.63; 1.99
ICU use (yes vs. no)	3.79	3.75; 3.84	3.88	3.69; 4.08	4.33	4.21; 4.45	3.51	3.46; 3.57	4.20	4.09; 4.32	4.62	4.46; 4.79
Ventilatory support use (ref.: no)												
Invasive	8.14	8.02; 8.27	9.56	8.99; 10.17	7.83	7.55; 8.13	7.11	6.96; 7.27	11.37	10.91; 11.84	9.83	9.38; 10.30
Non-invasive	1.14	1.13; 1.15	1.07	1.02; 1.12	0.91	0.88; 0.93	1.12	1.10; 1.14	1.64	1.58; 1.69	1.12	1.08; 1.16
Length of stay (ref: ≥ 1 day)												
0 day	3.76	3.65; 3.87	3.46	3.11; 3.85	4.15	3.86; 4.47	3.59	3.45; 3.73	4.24	3.92; 4.59	3.44	3.08; 3.84
Unknown	0.19	0.18; 0.19	0.09	0.08; 0.10	0.36	0.35; 0.38	0.12	0.11; 0.13	0.20	0.18; 0.23	0.25	0.23; 0.28
Patient residence city’s HDI (ref: very low/low (<0.600))												
Medium (0.600-0.699)	1.01	0.97; 1.04	0.99	0.91; 1.08	1.04	1.00; 1.08	0.87	0.75; 1.00	0.54	0.36; 0.79	1.23	0.89; 1.69
High (0.700-0.7999)	0.91	0.88; 0.94	0.87	0.80; 0.95	0.89	0.86; 0.93	0.79	0.69; 0.92	0.51	0.35; 0.75	1.18	0.86; 1.62
Very high (≥0.800)	0.86	0.83; 0.89	0.41	0.18; 0.93	1.16	0.78; 1.72	0.75	0.65; 0.87	0.45	0.31; 0.66	1.01	0.72; 1.40
Inpatient care unit’s COVID-19 hospitalizations ref: 100-299)												
300-599	0.71	0.64; 0.78	0.63	0.45; 0.89	0.62	0.48; 0.82	0.75	0.65; 0.87	0.70	0.57; 0.86	0.79	0.59; 1.05
600-999	0.56	0.50; 0.63	0.77	0.48; 1.24	0.53	0.39; 0.72	0.58	0.49; 0.69	0.59	0.47; 0.74	0.53	0.37; 0.76
1000-3999	0.44	0.39; 0.49	0.55	0.34; 0.91	0.42	0.31; 0.57	0.43	0.36; 0.50	0.50	0.39; 0.63	0.52	0.38; 0.72
≥ 4000	0.39	0.28; 0.55	0.37	0.13; 1.07	0.24	0.09; 0.68	0.46	0.28; 0.74	0.60	0.31; 1.18	0.38	0.17; 0.89
Healthcare unit type (ref: general hospital)												
Specialized hospital	0.73	0.60; 0.89	0.68	0.40; 1.18	0.65	0.44; 0.98	0.86	0.59; 1.27	0.71	0.41; 1.23	0.73	0.46; 1.17
Mixed unit	0.53	0.35; 0.81	0.50	0.17; 1.43	0.28	0.11; 0.72	0.66	0.38; 1.17				
General emergency center	1.65	1.23; 2.33			2.08	0.89; 4.86	1.33	0.92; 1.93			1.68	0.75; 3.75
Specialized emergency center	1.81	1.03; 3.17			1.67	0.51; 5.53	1.94	0.94; 4.01	0.59	0.13; 2.63		
Country region (ref: Southeast)												
North	1.18	1.01; 1.37										
Northeast	0.99	0.88; 1.10										
South	0.70	0.63; 0.78										
Midwest	0.85	0.74; 0.97										
Northeast/Southeast												
Inpatient care unit out of patient’s residence city (ref.: in)	1.04	1.02; 1.05	1.10	1.02; 1.18	1.13	1.09; 1.16	1.03	1.01; 1.05	0.99	0.96; 1.02	1.05	1.00; 1.11
Inpatient care unit’s city size (ref: <50,000 inhabitants)												
50,000-99,999 inhabitants	1.57	1.37; 1.79	1.65	1.08; 2.52	1.47	1.00; 2.16	1.51	1.22; 1.86	1.52	1.21; 1.90	1.71	1.13; 2.60
100,000-999,999 inhabitants	1.70	1.51; 1.91	1.30	0.88; 1.92	1.65	1.16; 2.35	1.80	1.51; 2.15	1.54	1.23; 1.93	1.43	1.03; 1.99
≥ 1,000,000 inhabitants	1.70	1.49; 1.94	1.68	1.07; 2.63	2.11	1.46; 3.05	1.52	1.23; 1.86	1.75	1.29; 2.36	1.08	0.75; 1.55
Pandemic period (ref: Sep-Nov 2020)												
Feb-May 2020 (wave 1.1)	1.33	1.30; 1.36	2.29	2.13; 2.46	1.76	1.68; 1.85	1.11	1.08; 1.14	0.73	0.66; 0.81	1.16	1.04; 1.30
Jun-Aug 2020 (wave 1.2)	1.05	1.03; 1.06	1.18	1.10; 1.28	1.25	1.20; 1.31	0.95	0.93; 0.98	0.94	0.90; 0.98	1.36	1.29; 1.43
Dec 2020 – Feb 2021 (wave2.1)	1.23	1.21; 1.25	2.22	2.08; 2.37	1.38	1.32; 1.44	1.07	1.04; 1.09	1.34	1.29; 1.39	1.13	1.07; 1.19
Mar 2021 – Apr 2021 (wave2.2)	1.63	1.60; 1.65	1.85	1.73; 1.98	1.66	1.60; 1.73	1.54	1.51; 1.57	1.77	1.71; 1.83	1.73	1.65; 1.82
May 2021 – Jun 2021 (wave2.3)	1.34	1.32; 1.37	1.33	1.21; 1.46	1.28	1.22; 1.35	1.31	1.28; 1.35	1.49	1.42; 1.55	1.30	1.22; 1.39
Jul 2021 – Dec 2021	0.86	0.85; 0.88	0.76	0.69; 0.83	0.85	0.81; 0.90	0.85	0.83; 0.87	0.91	0.87; 0.94	0.83	0.78; 0.87
Jan 2022 – Feb 2022 (wave3)	0.80	0.78; 0.82	0.67	0.61; 0.75	0.78	0.73; 0.82	0.81	0.79; 0.84	0.83	0.79; 0.88	0.68	0.63; 0.74
Mar 2022 – Dec 2022	0.51	0.50; 0.53	0.41	0.35; 0.48	0.48	0.45; 0.52	0.48	0.46; 0.49	0.65	0.61; 0.68	0.43	0.39; 0.47

Source: SIVEP Gripe - Sistema de Informação de Vigilância Epidemiológica da Gripe

The study excluded inpatient care units with less than 100 COVID-19 hospitalizations in the period

Data did not include neither general, nor specialized emergency centers in the North; neither mixed units, nor general emergency centers in the South; neither mixed units, nor specialized emergency centers in the Midwest

The findings in Table 4 indicate that there was variability among the regions of the country concerning the effects of the inpatient care unit categories defined based on ownership and participation in the SUS. Brazil mirrors, to some extent, the volume of hospitalizations observed in the Southeast, as it presents a similar standard of results to that registered in the region. For the country, the odds of COVID-19 inpatient mortality, compared to SUS public hospitals, were 11% lower in SUS philanthropic hospitals, 42% lower in non-SUS public hospitals, 43% lower in SUS private hospitals, 57% lower in non-SUS private hospitals, and 62% lower in non-SUS philanthropic hospitals. In all macro-regions, the odds of inpatient mortality were lower (protective effect) for non-SUS private hospitals, and except for the Northeast, they were also lower for non-SUS philanthropic hospitals. Non-SUS public hospitals performed better than SUS public units in the Northeast and the Southeast, SUS private hospitals in the Northeast, Southeast and Midwest, and the SUS philanthropic hospitals only in the Southeast.

The results also indicate statistically significant increase in the odds of inpatient mortality as a function of age, for males, blacks (in Brazil and all regions, except for the North), and indigenous individuals (in Brazil, in the North, and in the Midwest – borderline significant) (Table 4). Unexpectedly, the odds of inpatient mortality for indigenous individuals in Southeastern Brazil was significantly lower. Still looking at race/color, we explored a specific categorization (Supplement 4) to highlight the higher odds of death for mixed race and Asian, in addition to black individuals, in the South, as indicated in Table S3.1. Unknow race did not differ significantly from white individuals in relation to the risk of inpatient death in that region, and these findings sound consistent and relevant in face of the very distinct racial distribution in the South, with large majority of whites.

Despite the gradient indicating the reduction in inpatient mortality with more years of education, in the bivariate analyses, the elevated unknown data for the variable, more than three times as much as that for race/color, did not allow for its inclusion in the multivariate models (Supplement 3 – Tables S3.1, S3.3, S3.4 and S3.5). The odds of mortality, additionally, increased with the number of comorbidities presented by the patient and with the presence of specific comorbidities, among which immunodepression must be highlighted.

Using the ICU, invasive ventilatory support and having a length of stay lower than a day were significantly and consistently associated with higher odds of inpatient mortality. Noninvasive ventilatory support, in turn, was associated with the reduction in the mortality risk in the Northeast and with its elevation in the other regions. Although 87.5% of the patients who received invasive ventilatory support were attended in an ICU, they correspond to less than half (45.8%) of all patients who received intensive care. Both variables kept high significant independent effects in the models.

The different types of healthcare units were not present in all regions. The mixed units were associated with a decrease in the inpatient mortality risk in Brazil and in the Northeast, while the general and specialized emergency centers were associated with an increase in the odds of mortality only in the overall analysis for the country. Other factors being constant, in general, the odds of COVID-19 inpatient mortality were lower for patients living in cities with higher HDI and did not present a clear common pattern related to the increase in the size of the municipalities. In Brazil, as a whole, in the North, Northeast and Southeast, the odds of inpatient mortality were statistically higher for patients who were hospitalized out of their residence’s municipality.

Among the macro-regions in Brazil, the North demonstrated the poorest performance, as previously indicated, while the South exhibited the best performance. The Midwest region was also found to be associated with a reduction in the odds of mortality compared to the Southeast, the reference category, while the Northeast and the Southeast, did not differentiate statistically from each other.

Finally, Table 4 shows the pandemic dynamics in Brazil, indicating the highest adjusted odds of inpatient mortality between March and April 2021 in the country and in the Southeast, South and Midwest. The North was more heavily affected during the first phase of the first wave, especially between April and May 2020, and in the second wave, from December 2020 to February 2021. The Northeast also presented very high odds of inpatient mortality before June 2020. From the second semester of 2021 ahead, the odds of inpatient mortality consistently decreased, even with the third wave in January and February 2022. Compared to the period of lower inpatient mortality in 2020 (September-November), the odds of inpatient mortality decreased from March 2022 by 49% in Brazil, 59% in the North, 52% in the Northeast, 52% in the Southeast, 35% in the South and 57% in the Midwest.

## Discussion

Surpassing the various roles played by the SUS in the general response to the COVID-19 pandemic, including functions such as epidemiological surveillance, primary healthcare, and vaccination, among others, one of the findings of this study underscored, as expected, its critical importance to inpatient care delivery. More than 70% of the COVID-19 hospitalizations in Brazil took place in the public system between February 2020 and December 2022. However, the findings also suggest weaknesses in the performance of the SUS inpatient care units compared to the private sector or even the non-SUS public inpatient care units, reflecting accumulated structural and financing problems [40]. Significant differences in inpatient care mortality were identified among inpatient care unit categories defined based on ownership and participation in the SUS. Non-SUS philanthropic and private hospitals had the best rates of inpatient mortality in the country, while the worst inpatient mortality outcomes were observed for SUS public hospitals, particularly in the Northeast and Southeast regions. Overall, the South region performed better than other regions in terms of the outcome. Northern Brazil had the highest likelihood of inpatient mortality.

This study ratifies findings from other studies carried out at the beginning of the pandemic, stressing a higher risk of death associated with increasing age, male sex, presence of comorbidities, use of ICUs and use of invasive ventilatory support [27, 28]. It confirmed high inpatient mortality within the first 24 hours of hospitalization, which likely reflected underlying healthcare access issues [27].

It also found that black individuals had higher chances of COVID-19 inpatient mortality [26, 27] in all regions of the country, except for the North, as well as indigenous individuals in the North [41], and in the Midwest (borderline significant). The elevated level of missing values, proportionally higher in non-SUS units, compromised the possibility of detecting expected differences between mixed raced and white persons, except for Southern Brazil, where supplementary results indicated higher odds of death for mixed race and Asian individuals, compared to whites. On the one hand, these findings are concerning and highlight systemic inequities that exist in the country; on the other hand, they also reflect problems that persist in the assessment of health inequities by race, based on available data. Beyond the high frequency of missing values, racial measurement in Brazil has referred to phenotype (physical appearance) and not to ancestry (origin) [42], there existing the recommendation that race/skin color should be self-declared, what may incur a subjective judgement affectable by contextual aspects and able to produce variability in the categories. The mixed race group may be especially heterogeneous. The fact that the data mostly did not allow for differentiation of the risk of COVID-19 inpatient mortality between mixed race and white individuals in this study do not mean necessarily that differences do not exist. Nevertherless, the aggregation of both in the reference category sounded more adjusted to the data pattern [42], than the more conventional and frequent aggregation of it with blacks. However, studies have shown that racial and ethnic minorities have experienced disproportionate impacts of the COVID-19 pandemic, which can be attributed to a range of factors, including higher rates of underlying health conditions, occupation-related exposure, socioeconomic adverse conditions, and unequal access to healthcare [26, 41]. It is also interesting to highlight regional variations in the impact of the pandemic across more vulnerable groups, with a note on the lower odds of inpatient mortality among indigenous found in the Southeast, probably reflecting a very different context regarding healthcare access. The findings suggest that there may be a complex interplay between race, ethnicity, geography, and other social determinants of health that contributed to the disparities in COVID-19 outcomes in Brazil. To address these disparities, which also affect other health conditions, there is a need for a more comprehensive approach that accounts for the social determinants of health. This includes ensuring that all individuals have access to affordable and equitable healthcare, as well as providing targeted public health interventions to reduce the impact of health problems on vulnerable populations.

Over time, inpatient mortality presented slightly different standards in the macro-regions, with higher rates in the first months of the pandemics in 2020, and, more spread, in the first semester of 2021, following a true disaster in the North between December 2020 and January 2021. It was more critical when the healthcare system was under heavy load and pressure by COVID-19 [19, 25, 38, 43]. Overall, considering the conjunction of very high volume of COVID-19 hospitalizations and inpatient mortality rate, the death occurrence peak took place in March 2021, when, in the whole country, hospitals were at or above capacity, leading to shortages of critical resources such as ventilators, oxygen, and ICU beds [44].

The COVID-19 pandemic has resulted in an unprecedented strain on Brazil's healthcare system, with a surge in hospitalizations and deaths. Vaccination has proven to be a crucial tool in reducing the burden on the healthcare system. This study showed a consistent reduction in the odds of COVID-19 inpatient mortality from the second semester of 2021, when vaccination in Brazil reached broader coverage. The wave related to the Omicron variant in the beginning of 2022 led to a modest surge in hospitalizations, but the less aggressive characteristics of the variant combined with vaccination levels allowed for the sustained declining trend in the odds of inpatient mortality. Therefore, our findings are compatible with those of other studies that have shown that COVID-19 vaccination is associated with a significant reduction in hospitalization and inpatient mortality rates. Here, we underline two Brazilian studies that found a significant reduction in severe COVID-19 cases, hospitalizations and deaths of elderly individuals after vaccination when it was still restricted to more vulnerable groups, thus producing important declines in relative mortality compared to younger individuals [36, 45], and another study that provided evidence of the effectiveness of COVID-19 vaccination, including during the Omicron wave [32].

This study also found that hospitals with a higher volume of COVID-19 patients in the whole period of analysis tended to have better outcomes. This suggests that there may be a learning curve effect as hospitals gain more experience in treating COVID-19 patients, so hospitals that have treated more COVID-19 patients would likely have more adequate and sufficient physical infrastructure and workforce, developed protocols for patient care, better understanding of potential complications, and thereby more experienced staff. On the one hand, the findings were consistent with previously published research that showed volume-outcome relationships across various medical conditions [46]. On the other hand, it contrasted with the results of a study carried out in 85 hospitals in the United States of America in which there was no significant association of in-hospital case-fatality rate with overall hospital COVID-19 case volume but rather with more rapid COVID-19 case-growth [47]. Other studies indicated, in specific periods, high COVID-19 hospital prevalence as a risk factor for mortality [48, 49]. It is important to note that to provide high-quality care, hospitals need resources, investments, teamwork, and expertise.

Living in cities with higher HDI and being assisted in inpatient care units in the residence municipality [27, 29] were found to be independently protective against the risk of COVID-19 inpatient mortality, and there are regional differences among the effects of being transferred to be treated out of the own municipality. The odds of inpatient mortality were found to increase 10% and 13% among those who were hospitalized out of their residence cities in the North and the Northeast, respectively. In the South, it made no difference to be treated in another city. These differences may reflect more concentrated use of healthcare resources rather than a spread one, with repercussions on travel time to adequate care access. It is also interesting to note that this study did not find a difference between the effects of being transferred to a city in the same health region or out of it.

The pandemic was an opportunity for the SUS to show its magnitude and strength by providing care in all regions of the country [50–52]. Nevertheless, it has also been a pivotal point to show areas where the system could improve. In a context of huge regional variation, in which SUS and non-SUS, public, private, and philanthropic inpatient care providers coexist and have different levels of investment, it is crucial to tackle growing inequities in access to high-quality healthcare. The lower performance of the SUS, which covered more than 70% of the COVID-19 hospitalizations and ordinarily also provides healthcare to the majority of the Brazilian population exclusively reliant on it, represents a major issue that must be addressed. The Brazilian universal and public system needs to be strengthened, requiring more funds and capital investment, through an agenda that must support sustainable actions to improve healthcare structures, workforce, information systems and research to understand the drivers of health outcomes, including the role of demographic and socioeconomic factors, as well as the distribution of healthcare services.

This study has the strength of relying on a large dataset of COVID-19 hospitalizations in Brazil, including the public and private healthcare sectors, from February 2020 to December 2022 and aggregating over 1.6 million observations over that period. It also has the potency of having allowed for analyses that incorporated individual, inpatient care unit and municipality levels by combining data from SIVEP-Gripe with data from CNES and IBGE. Overall, the results provide a good overview of COVID-19 hospitalizations in relation to their occurrence over time, profiles of hospitalized patients, inpatient care characteristics and inpatient care mortality. Nevertheless, we recognize limitations regarding the accuracy, completeness, and real coverage of the secondary data utilized. In general, the quality of data may normally vary depending on the level of resources and training available to the staff responsible for reporting the information and was very likely exacerbated in the midst of the chaos presented by the pandemic and the need to respond to all challenges presented.

SIVEP Gripe should encompass all COVID-19 hospitalizations in the public and private sectors in the country, but real coverage may compromise that expectation. Problems in the completeness of variables are also present and impose some caution in the interpretation of results. Moreover, the way comorbidities are registered does not allow for the utilization of classic case severity indexes such as the Charlson Comorbidity and Elixhauser Comorbidity, which would permit better accounting for multimorbidity and comparisons with other studies [27, 53, 54]. Under these conditions, the difficulties in assessing hospitals’ performance, controlling satisfactorily for case severity and social vulnerability of patients attended, and differentiate these factors from others related to the characteristics of the care units themselves are inexorable. Anyway, findings of this study seem majorly consistent, and indicate that higher COVID-19 inpatient mortality rates at SUS hospitals reflected not only the more vulnerable patients’ profile, but also healthcare quality problems.

## Conclusions

This work shows that COVID-19 inpatient mortality was affected, in addition to case severity, by sociodemographic and healthcare access, appropriateness and effectiveness inequities, highlighting problems in the SUS inpatient healthcare delivery performance. While SUS may face significant challenges and grapple with some specific outcomes, its many strengths make it an essential, unique, and valuable resource for Brazilians. Despite historical shortcomings, the SUS’ role during the pandemic highlighted its value for the health and healthcare equity. However, it is central to recognize its limits and, performance problems, in order to promote substantive improvement interventions.

Our results stress the need to invest and improve the system, especially targeting the causes of inequalities in supply, access, and outcomes. It also provides elements for the debate on the role and performance of each type of hospital care provider (private and public) in the Brazilian health system. Further research should investigate the underlying factors that explain the differences in inpatient mortality among hospitals, including their capacity to provide adequate medical resources and intensive care, their workforce, and their adherence to clinical protocols. Despite macro context determinants, changes, investments, and monitoring are necessary to avoid the risks of compromising universal access to health services and widening inequalities between SUS and non-SUS users. Measures such as investing in more healthcare infrastructure, increasing the number of healthcare professionals, providing better training and support for those workers as well as better wages and working conditions are fundamental. Policies in that direction could guarantee that the system is better equipped to handle crises and protect the health of the population.

As a consequence of COVID-19, new and old challenges are entangled, such as timely response to unmet needs and assurance of the sustainability and resilience of the universal public health system. This study provided useful insights into the variations in COVID-19 inpatient mortality in Brazil and highlighted the need for continued efforts to improve the quality and equity of healthcare for all.

### Supplementary Information

**Additional file 1. **COVID-19 inpatient mortality in Brazil from 2020 to 2022: a cross-sectional overview study based on secondary data. STROBE checklist.

**Additional file 2: Supplement 2.** Public inpatient healthcare units, discharges, and COVID-19 inpatient mortality.

**Additional file 3: Supplement 3.** Explorations on the inclusion of the variables “race” and “education”, and the exclusion of the variables “ICU”, “ventilatory support”, and “length of stay”. **Table S3.1.** Factors associated with COVID-19 inpatient mortality in Brazil and in the country’s macro-regions. Generalized linear mixed models with all categories of race and education and the variables ICU, ventilatory support and length of stay. Brazil, Feb 2020 – Dec 2022. **Table S3.2.** Factors associated with COVID-19 inpatient mortality in Brazil and in the country’s macro-regions. Generalized linear mixed models with all categories of race and education, and excluding the variables ICU use, ventilatory support use and length of stay. Brazil, Feb 2020 – Dec 2022. **Table S3.3.** Distribution of individuals by race/color in Brazil and macro-regions. **Table S3.4.** Distribution of individuals by education level in Brazil and macro-regions. **Table S3.5.** Distribution of known and unknown data on race/color and education level by inpatient care unit categories in Brazil and macro-regions.

**Additional file 4: Supplement 4.** Generalized linear mixed models: factors associated with COVID-19 inpatient mortality in Southern Brazil, Feb 2020 – Dec 2022.

## Data Availability

All data and the SAS^®^ program used in the study are available – Portela, Margareth (2023) , “COVID-19 inpatient mortality in Brazil from 2020 to 2022”, Mendeley Data, V1, https://doi.org/10.17632/d6cf479msc.1.
