# Supplementary material for: COVID-19 inpatient mortality in Brazil from 2020 to 2022: a cross-sectional overview study based on secondary data
Source: Int J Equity Health. 2023 Nov 17;22:238. doi: 10.1186/s12939-023-02037-8 (PMC10655483; doi:10.1186/s12939-023-02037-8)
Supplement: Supplementary file 2 — Additional file 2: Supplement 2. Public inpatient healthcare units, discharges, and COVID-19 inpatient mortality. [file 12939_2023_2037_MOESM2_ESM.docx]

**Supplement 2**

Public inpatient healthcare units, discharges, and COVID-19 inpatient mortality

| Government  level | Public SUS | | | | | Public non-SUS | | | | |
| --- | --- | --- | --- | --- | --- | --- | --- | --- | --- | --- |
|  | Inpatient care units | Discharges | Inpatient mortality (%) | | | Inpatient care units | Discharges | Inpatient mortality (%) | | |
|  |  |  | Proportion | STD | 95% CI |  |  | Proportion | STD | 95% CI |
| Federal | 31  (3,5%) | 20,324  (3.2%) | 38.2 | 48.6 | 37.5; 38.9 | 13  (1.5%) | 6,881  (1.1%) | 35.7 | 47.9 | 34.6; 36.9 |
| State | 346  (38.5%) | 312,669  (48.4%) | 37.6 | 48.4 | 37.4; 37.8 | 8  (0.9%) | 6,205  (1.0%) | 28.5 | 45.1 | 27.3; 28.6 |
| Municipal | 498  (55.4% | 297,330  (46.1%) | 37.3 | 48.4 | 37.1; 37.4 | 2  (0.2%) | 513  (0.1% | 30.2 | 46.0 | 26.2; 34.2 |
| Public Consortium (Public Association) | 1  (0.1%) | 1,701  (0.3%) | 33.9 | 47.3 | 31.6; 36.1 |  |  |  |  |  |

Among the public hospitals with COVID-19 hospitalizations, 97.5% were related to the SUS. Municipal SUS units corresponded to the majority (55.4%), comprising 46.1% of the discharges, and was followed by the state SUS units (38.5%), responsible for 48.4 of the discharges. The participation of federal units was much lower.
