# Supplementary material for: COVID-19 inpatient mortality in Brazil from 2020 to 2022: a cross-sectional overview study based on secondary data
Source: Int J Equity Health. 2023 Nov 17;22:238. doi: 10.1186/s12939-023-02037-8 (PMC10655483; doi:10.1186/s12939-023-02037-8)
Supplement: Supplementary file 3 — Additional file 3: Supplement 3. Explorations on the inclusion of the variables “race” and “education”, and the exclusion of the variables “ICU”, “ventilatory support”, and “length of stay”. Table S3.1. Factors associated with COVID-19 inpatient mortality in Brazil and in the country’s macro-regions. Generalized linear mixed models with all categories of race and education and the variables ICU, ventilatory support and length of stay. Brazil, Feb 2020 – Dec 2022. Table S3.2. Factors associated with COVID-19 inpatient mortality in Brazil and in the country’s macro-regions. Generalized linear mixed models with all categories of race and education, and excluding the variables ICU use, ventilatory support use and length of stay. Brazil, Feb 2020 – Dec 2022. Table S3.3. Distribution of individuals by race/color in Brazil and macro-regions. Table S3.4. Distribution of individuals by education level in Brazil and macro-regions. Table S3.5. Distribution of known and unknown data on race/color and education level by inpatient care unit categories in Brazil and macro-regions. [file 12939_2023_2037_MOESM3_ESM.docx]

**Supplement 3**

**Explorations on the inclusion of the variables “race” and “education”, and the exclusion of the variables “ICU”, “ventilatory support”, and “length of stay”**

Here we provide intermediary explorations on models including all categories of the socioeconomic variables “education” and “race” (Tables S3.1 and S3.2). It was observed an expressive protective effect of unknown race/color and education level, what makes little sense, but, indicates a non-aleatory pattern of data sub-registration. Description of missing data of these variables by macro-regions and healthcare unit category are also presented in Tables S3.3-S3.5. Missing data in education variable are quite superior (64,1%, range 51,5-75.2 across macro-regions) to those in race (18.6%, range 6.8-27.1 across macro-regions). These results and the missing data volume supported the decision to exclude "education" from the final models. Therefore, in the final models, only “race” was used as a socioeconomic variable.

Concerning the supply, process of care variables and severity case relationship, models excluding ICU use, length of stay and ventilatory support were presented in Table S3.2. Differences of the effects related to the structure/ complexity of the hospitals on mortality tended to vanish, impacting on (1) the increase in the odds ratios of case severity variables (age and comorbidities); (2) the almost disappearance of regional differences; (3) reduction of differences among inpatient care unit categories, and (4) increase in the odds ratios related to the hospital location in larger cities. Thus, the final models (Table 4) included ICU use, ventilatory support use, and the length of stay as independent variables. In some degree these healthcare process measures express the case severity, but also differentiate the complexity of the hospital supply available, which, itself, is a relevant aspect in considering the healthcare quality provided.

**Table S3.1. Factors associated with COVID-19 inpatient mortality in Brazil and in the country’s macro-regions. Generalized linear mixed models with all categories of race and education and the variables ICU, ventilatory support and length of stay. Brazil, Feb 2020 – Dec 2022.**

| Variable | Brazil | | North | | Northeast | | Southeast | | South | | Midwest | |
| --- | --- | --- | --- | --- | --- | --- | --- | --- | --- | --- | --- | --- |
|  | OR | 95%CI | OR | 95%CI | OR | 95%CI | OR | 95%CI | OR | 95%CI | OR | 95%CI |
| Inpatient healthcare unit category (ref: Public SUS) |  |  |  |  |  |  |  |  |  |  |  |  |
| Public non-SUS | 0.58 | 0.45; 0.74 | 0.42 | 0.14; 1.32 | 0.34 | 0.18; 0.66 | 0.59 | 0.42; 0.83 | 0.65 | 0.34; 1.24 | 0.99 | 0.53; 1.85 |
| Private SUS | 0.57 | 0.47; 0.69 | 0.86 | 0.47; 1.57 | 0.52 | 0.35; 0.78 | 0.42 | 0.27; 0.66 | 1.02 | 0.67; 1.56 | 0.56 | 0.40; 0.80 |
| Private non-SUS | 0.43 | 0.39; 0.48 | 0.64 | 0.39; 1.05 | 0.43 | 0.32; 0.58 | 0.36 | 0.31; 0.42 | 0.61 | 0.46; 0.82 | 0.52 | 0.38; 0.71 |
| Philanthropic SUS | 0.87 | 0.78; 0.97 | 0.75 | 0.41; 1.37 | 0.96 | 0.71; 1.29 | 0.68 | 0.58; 0.80 | 1.20 | 0.97; 1.48 | 1.05 | 0.76; 1.46 |
| Philanthropic non-SUS | 0.37 | 0.30; 0.46 | 0.45 | 0.21; 0.95 | 0.94 | 0.37; 2.41 | 0.33 | 0.25; 0.44 | 0.39 | 0.25; 0.62 | 0.34 | 0.18; 0.62 |
| Age (ref: 18-39 years) |  |  |  |  |  |  |  |  |  |  |  |  |
| 40-49 years | 1.45 | 1.42; 1.48 | 1.65 | 1.53; 1.77 | 1.42 | 1.35; 1.49 | 1.43 | 1.39; 1.47 | 1.48 | 1.41; 1.55 | 1.47 | 1.38; 1.56 |
| 50-59 years | 2.10 | 2.06; 2.14 | 2.52 | 2.35; 2.69 | 2.00 | 1.91; 2.09 | 2.09 | 2.04; 2.15 | 2.16 | 2.06; 2.26 | 1.99 | 1.88; 2.10 |
| 60-69 years | 3.46 | 3.40; 3.53 | 4.36 | 4.07; 4.66 | 3.14 | 3.00; 3.28 | 3.49 | 3.40; 3.58 | 3.58 | 3.42; 3.74 | 3.25 | 3.07; 3.44 |
| 70-79 years | 5.65 | 5.55; 5.76 | 6.95 | 6.48; 7.47 | 4.90 | 4.69; 5.13 | 5.66 | 5.51; 5.81 | 6.47 | 6.18; 6.78 | 4.99 | 4.70; 5.29 |
| ≥ 80 years | 10.98 | 10.77; 11.20 | 10.63 | 9.85; 11.48 | 8.71 | 8.32; 9.13 | 11.11 | 10.80; 11.43 | 14.79 | 14.08; 15.54 | 9.29 | 8.73; 9.90 |
| Male (yes vs. no) | 1.21 | 1.20; 1.22 | 1.15 | 1.11; 1.19 | 1.14 | 1.12; 1.17 | 1.22 | 1.21; 1.24 | 1.22 | 1.20; 1.25 | 1.23 | 1.20; 1.27 |
| Race/color (ref: white) |  |  |  |  |  |  |  |  |  |  |  |  |
| Black | 1.07 | 1.04; 1.09 | 0.93 | 0.82; 1.05 | 0.94 | 0.88; 1,01 | 1.07 | 1.04; 1.10 | 1.09 | 1.02; 1.16 | 1.11 | 1.02; 1.21 |
| Mixed race | 0.90 | 0.89; 0.91 | 0.89 | 0.84; 0.95 | 0.76 | 0.74; 0.79 | 0.90 | 0.89; 0.92 | 1.11 | 1.06; 1.17 | 0.99 | 0.95; 1.03 |
| Asian | 0.94 | 0.90; 0.98 | 0.85 | 0.71; 1.03 | 0.73 | 0.65; 0.81 | 0.90 | 0.83; 0.96 | 1.27 | 1.10; 1.46 | 1.10 | 0.97; 1.25 |
| Indigenous | 1.06 | 0.95; 1.18 | 1.42 | 1.19; 1.71 | 0.73 | 0.54; 0.98 | 0.61 | 0.47; 0.80 | 1.16 | 0.84; 1.60 | 1.25 | 0.96; 1.63 |
| Unknown | 0.75 | 0.73; 0.76 | 0.67 | 0.61; 0.74 | 0.65 | 0.62; 0.68 | 0.67 | 0.66; 0.69 | 1.01 | 0.96; 1.05 | 1.02 | 0.97; 1.07 |
| Education (ref: no schooling/primary) |  |  |  |  |  |  |  |  |  |  |  |  |
| Lower secondary | 1.00 | 0.98; 1.02 | 0.92 | 0.86; 0.99 | 1.02 | 0.96; 1.09 | 1.03 | 1.01; 1.06 | 0.91 | 0.87; 0.95 | 0.99 | 0.92; 1.07 |
| Upper secondary | 0.95 | 0.93; 0.97 | 0.80 | 0.75; 0.86 | 0.98 | 0.93; 1,04 | 1.00 | 0.97; 1.02 | 0.87 | 0.83; 0.91 | 0.85 | 0.80; 0.92 |
| Tertiary | 0.91 | 0.89; 0.94 | 0.85 | 0.78; 0.93 | 0.91 | 0.85; 0.99 | 0.98 | 0.93; 1.01 | 0.77 | 0.73; 0.82 | 0.80 | 0.74; 0.88 |
| Unknown | 0.65 | 0.64; 0.66 | 0.54 | 0.51; 0.57 | 0.85 | 0.82; 0.89 | 0.55 | 0.54; 0.56 | 0.80 | 0.78; 0.83 | 1.02 | 0.96; 1.08 |
| Comorbidities (ref: 0) |  |  |  |  |  |  |  |  |  |  |  |  |
| 1 | 1.29 | 1.27; 1.30 | 1.17 | 1.12; 1.22 | 1.26 | 1.23; 1.30 | 1.25 | 1.23; 1.27 | 1.55 | 1.50; 1.60 | 1.34 | 1.29; 1.39 |
| 2 | 1.47 | 1.45; 1.49 | 1.23 | 1.16; 1.29 | 1.36 | 1.31; 1.40 | 1.43 | 1.40; 1.46 | 1.84 | 1.78; 1.90 | 1.61 | 1.54; 1.68 |
| ≥ 3 | 1.66 | 1.63; 1.69 | 1.22 | 1.11; 1.33 | 1.39 | 1.33; 1.46 | 1.62 | 1.58; 1.66 | 2.20 | 2.11; 2.30 | 1.86 | 1.75; 1.98 |
| Down syndrome (yes vs. no) | 1.18 | 1.09; 1.28 | 1.33 | 0.98; 1.81 | 1.22 | 0.99; 1,49 | 1.32 | 1.17; 1.48 | 1.10 | 0.91; 1.33 | 0.79 | 0.58; 1.06 |
| Obesity (yes vs. no) | 1.18 | 1.16; 1.20 | 1.54 | 1.41; 1.68 | 1.10 | 1.06; 1.16 | 1.23 | 1.12; 1.26 | 1.04 | 1.01; 1.08 | 1.27 | 1.20; 1.35 |
| Hematologic disease (yes vs. no) | 1.25 | 1.19; 1.31 | 1.06 | 0.82; 1.39 | 1.22 | 1.06; 1.41 | 1.27 | 1.19; 1.36 | 1.32 | 1.18; 1.48 | 1.10 | 0.92; 1.32 |
| Hepatic disease (yes vs. no) | 1.65 | 1.58; 1.73 | 1.30 | 1.04; 1.62 | 1.86 | 1.66; 2.08 | 1.66 | 1.56; 1.77 | 1.67 | 1.51; 1.84 | 1.50 | 1.28; 1.75 |
| Neurologic disease (yes vs. no) | 1.62 | 1.59; 1.66 | 1.21 | 1.06; 1.38 | 1.41 | 1.32; 1.50 | 1.58 | 1.54; 1.63 | 1.91 | 1.83; 2.00 | 1.34 | 1.24; 1.46 |
| Pneumopathy (yes vs. no) | 1.25 | 1.22; 1.28 | 1.32 | 1.16; 1.51 | 1.12 | 1.05; 1.20 | 1.25 | 1.21; 1.29 | 1.24 | 1.19; 1.30 | 1.20 | 1.11; 1.30 |
| Kidney disease (yes vs. no) | 1.55 | 1.51; 1.58 | 1.51 | 1.36; 1.68 | 1.59 | 1.50; 1.68 | 1.55 | 1.51; 1.60 | 1.51 | 1.43; 1.59 | 1.56 | 1.44; 1.68 |
| Immunodepression (yes vs. no) | 1.96 | 1.91; 2.01 | 1.87 | 1.64; 2.14 | 1.92 | 1.78; 2,06 | 1.91 | 1.84; 1.98 | 2.25 | 2.12; 2.38 | 1.80 | 1.64; 1.99 |
| ICU use (yes vs. no) | 3.81 | 3.77; 3.85 | 3.89 | 3.70; 4.09 | 4.32 | 4.20; 4.44 | 3.54 | 3.49; 3.59 | 4.21 | 4.09; 4.32 | 4.62 | 4.46; 4,79 |
| Ventilatory support use (ref.: no) |  |  |  |  |  |  |  |  |  |  |  |  |
| Invasive | 8.09 | 7.97; 8.22 | 9.23 | 8.67; 9.82 | 7.75 | 7.47; 8.05 | 7.13 | 6.98; 7.28 | 11.39 | 10.93; 11.86 | 9.88 | 9.43; 10.36 |
| Non-invasive | 1.13 | 1.11; 1.14 | 1.05 | 1.00; 1.09 | 0.90 | 0.88; 0.93 | 1.11 | 1.09; 1.12 | 1.64 | 1.58; 1.69 | 1.13 | 1.08; 1.17 |
| Length of stay (ref: ≥ 1 day) |  |  |  |  |  |  |  |  |  |  |  |  |
| 0 day | 3.72 | 3.61; 3.83 | 3.52 | 3.17; 3.92 | 4.14 | 3.85; 4.46 | 3.49 | 3.35; 3.63 | 4.24 | 3.91; 4.59 | 3.44 | 3.08; 3.85 |
| Unknown | 0.19 | 0.18; 0.20 | 0.09 | 0.08; 0.10 | 0.37 | 0.35; 0.39 | 0.12 | 0.12; 0.13 | 0.20 | 0.18; 0.23 | 0.25 | 0.23; 0.28 |
| Patient residence city’s HDI (ref: very low/Low (<0.600)) |  |  |  |  |  |  |  |  |  |  |  |  |
| Medium (0.600-0.699) | 1.01 | 0.98; 1.04 | 1.00 | 0.92; 1.08 | 1.04 | 1.00; 1.08 | 0.86 | 0.73; 0.99 | 0.54 | 0.37; 0.79 | 1.24 | 0.90; 1.70 |
| High (0.700-0.7999) | 0.91 | 0.88; 0.94 | 0.88 | 0.81; 0.95 | 0.89 | 0.85; 0.93 | 0.76 | 0.66; 0.88 | 0.51 | 0.35; 0.76 | 1.19 | 0.87; 1.63 |
| Very high (≥0.800) | 0.84 | 0.81; 0.88 | 0.43 | 0.19; 1.00 | 1.15 | 0.77; 1.71 | 0.70 | 0.61; 0.81 | 0.45 | 0.31; 0.67 | 1.02 | 0.73; 1.41 |
| Inpatient care unit’s COVID-19 hospitalizations (ref: 100-299) |  |  |  |  |  |  |  |  |  |  |  |  |
| 300-599 | 0.70 | 0.63; 0.78 | 0.61 | 0.43; 0.86 | 0.62 | 0.47; 0.82 | 0.74 | 0.63; 0.86 | 0.71 | 0.58; 0.87 | 0.79 | 0.59; 1.05 |
| 600-999 | 0.55 | 0.49; 0.62 | 0.76 | 0.46; 1.26 | 0.53 | 0.39; 0.72 | 0.56 | 0.47; 0.67 | 0.58 | 0.46; 0.74 | 0.53 | 0.37; 0.76 |
| 1000-3999 | 0.43 | 0.38; 0.48 | 0.55 | 0.33; 0.91 | 0.42 | 0.31; 0.57 | 0.42 | 0.35; 0.49 | 0.49 | 0.38; 0.62 | 0.51 | 0.37; 0.71 |
| ≥ 4000 | 0.39 | 0.28; 0.55 | 0.39 | 0.13; 1.18 | 0.26 | 0.09; 0.74 | 0.44 | 0.26; 0.73 | 0.60 | 0.30; 1.19 | 0.38 | 0.17; 0.88 |
| Healthcare unit type (ref: general hospital) |  |  |  |  |  |  |  |  |  |  |  |  |
| Specialized hospital | 0.70 | 0.57; 0.85 | 0.67 | 0.38; 1.18 | 0.64 | 0.43; 0.95 | 0.81 | 0.54; 1.22 | 0.71 | 0.41; 1.24 | 0.75 | 0.47; 1.20 |
| Mixed unit | 0.50 | 0.32; 0.77 | 0.42 | 0.14; 1.25 | 0.27 | 0.11; 0.71 | 0.60 | 0.33; 1.10 |  |  |  |  |
| General emergency center | 1.58 | 1.16; 2.14 |  |  | 2.10 | 0.89; 4.95 | 1.24 | 0.84; 1.84 |  |  | 1.72 | 0.77; 3.84 |
| Specialized emergency center | 1.82 | 1.02; 3.25 |  |  | 1.63 | 0.49; 5.44 | 1.89 | 0.88; 4.08 | 0.61 | 0.13; 2.76 |  |  |
| Country region |  |  |  |  |  |  |  |  |  |  |  |  |
| North (ref: Southeast) | 1.15 | 0.99; 1.34 |  |  |  |  |  |  |  |  |  |  |
| Northeast | 1.08 | 0.96; 1.21 |  |  |  |  |  |  |  |  |  |  |
| South | 0.68 | 0.61; 0.76 |  |  |  |  |  |  |  |  |  |  |
| Midwest | 0.89 | 0.77; 1.02 |  |  |  |  |  |  |  |  |  |  |
| Inpatient care unit out of patient’s residence city (ref.: in) | 1.04 | 1.03; 1.06 | 1.10 | 1.03; 1.18 | 1.13 | 1.09; 1.17 | 1.03 | 1.01; 1.05 | 0.99 | 0.96; 1.02 | 1.06 | 1.00; 1.12 |
| Inpatient care unit’s city size (ref: <50,000 inhabitants) |  |  |  |  |  |  |  |  |  |  |  |  |
| 50,000-99,999 inhabitants | 1.58 | 1.38; 1.81 | 1.64 | 1.06; 2.56 | 1.47 | 1.00; 2.17 | 1.55 | 1.24; 1.93 | 1.51 | 1.20; 1.90 | 1.69 | 1.11; 2.56 |
| 100,000-999,999 inhabitants | 1.81 | 1.61; 2.04 | 1.42 | 0.94; 2.14 | 1.69 | 1.19; 2.42 | 2.06 | 1.71; 2.49 | 1.55 | 1.24; 1.95 | 1.42 | 1.02; 1.97 |
| ≥ 1,000,000 inhabitants | 1.98 | 1.73; 2.26 | 1.90 | 1.19; 3.04 | 2.26 | 1.56; 3.27 | 1.90 | 1.53; 2.36 | 1.81 | 1.33; 2.46 | 1.04 | 0.72; 1.50 |
| Pandemic period (ref: Sep-Nov 2020) |  |  |  |  |  |  |  |  |  |  |  |  |
| Feb-May 2020 (wave 1.1) | 1.35 | 1.32; 1.38 | 2.33 | 2.16; 2.51 | 1.80 | 1.72; 1.88 | 1.14 | 1.11; 1.17 | 0.73 | 0.66; 0.81 | 1.17 | 1.04; 1.31 |
| Jun-Aug 2020 (wave 1.2) | 1.04 | 1.03; 1.06 | 1.18 | 1.09; 1.27 | 1.25 | 1.20; 1.31 | 0.95 | 0.92; 0.97 | 0.93 | 0.89; 0.97 | 1.36 | 1.29; 1.43 |
| Dec 2020 – Feb 2021 (wave2.1) | 1.23 | 1.22; 1.25 | 2.27 | 2.13; 2.42 | 1.38 | 1.33; 1.44 | 1.08 | 1.05; 1.10 | 1.34 | 1.29; 1.39 | 1.13 | 1.07; 1.19 |
| Mar 2021 – Apr 2021 (wave2.2) | 1.65 | 1.62; 1.67 | 1.87 | 1.74; 2.00 | 1.67 | 1.61; 1.74 | 1.57 | 1.54; 1.60 | 1.77 | 1.71; 1.83 | 1.73 | 1.65; 1.81 |
| May 2021 – Jun 2021 (wave2.3) | 1.36 | 1.33; 1.38 | 1.33 | 1.21; 1.46 | 1.29 | 1.23; 1.35 | 1.34 | 1.30; 1.37 | 1.50 | 1.43; 1.56 | 1.30 | 1.22; 1.38 |
| Jul 2021 – Dec 2021 | 0.87 | 0.85; 0.89 | 0.75 | 0.68; 0.82 | 0.85 | 0.81; 0.90 | 0.86 | 0.84; 0.88 | 0.91 | 0.87; 0.95 | 0.83 | 0.78; 0.87 |
| Jan 2022 – Feb 2022 (wave3) | 0.81 | 0.80; 0.83 | 0.66 | 0.60; 0.74 | 0.78 | 0.74; 0.83 | 0.85 | 0.82; 0.87 | 0.84 | 0.80; 0.89 | 0.68 | 0.63; 0.74 |
| Mar 2022 – Dec 2022 | 0.53 | 0.51; 0.54 | 0.40 | 0.35; 0.47 | 0.48 | 0.45; 0.52 | 0.49 | 0.48; 0.51 | 0.66 | 0.62; 0.69 | 0.43 | 0.39; 0.47 |

Source: SIVEP Gripe - Sistema de Informação de Vigilância Epidemiológica da Gripe.

The study excluded inpatient care units with less than 100 COVID-19 hospitalizations in the period.

**Table S3.2. Factors associated with COVID-19 inpatient mortality in Brazil and in the country’s macro-regions. Generalized linear mixed models with all categories of race and education, and excluding the variables ICU use, ventilatory support use and length of stay. Brazil, Feb 2020 – Dec 2022.**

| Variable | Brazil | | North | | Northeast | | Southeast | | South | | Midwest | |
| --- | --- | --- | --- | --- | --- | --- | --- | --- | --- | --- | --- | --- |
|  | OR | 95%CI | OR | 95%CI | OR | 95%CI | OR | 95%CI | OR | 95%CI | OR | 95%CI |
| Inpatient healthcare unit category (ref: Public SUS) |  |  |  |  |  |  |  |  |  |  |  |  |
| Public non-SUS | 0.55 | 0.43; 0.69 | 0.54 | 0.18; 1.65 | 0.33 | 0.17; 0.63 | 0.57 | 0.42; 0.78 | 0.56 | 0.27; 1.16 | 0.85 | 0.47; 1.54 |
| Private SUS | 0.74 | 0.61; 0.88 | 0.90 | 0.50; 1.63 | 0.74 | 0.51; 1.10 | 0.57 | 0.38; 0.85 | 0.93 | 0.58; 1.50 | 0.81 | 0.58; 1.12 |
| Private non-SUS | 0.51 | 0.46; 0.57 | 1.01 | 0.62; 1.62 | 0.49 | 0.36; 0.64 | 0.43 | 0.37; 0.50 | 0.60 | 0.43; 0.83 | 0.55 | 0.41; 0.74 |
| Philanthropic SUS | 0.92 | 0.83; 1.02 | 0.84 | 0.47; 1.51 | 0.89 | 0.67; 1.19 | 0.77 | 0.67; 0.89 | 1.08 | 0.85; 1.37 | 1.11 | 0.81; 1.52 |
| Philanthropic non-SUS | 0.47 | 0.38; 0.57 | 0.64 | 0.31; 1.32 | 0.58 | 0.23; 1.44 | 0.43 | 0.33; 0.55 | 0.62 | 0.37; 1.03 | 0.39 | 0.22; 0.70 |
| Age (ref: 18-39 years) |  |  |  |  |  |  |  |  |  |  |  |  |
| 40-49 years | 1.47 | 1.44; 1.49 | 1.63 | 1.54; 1.73 | 1.42 | 1.37; 1.48 | 1.44 | 1.40; 1.47 | 1.52 | 1.46; 1.58 | 1.46 | 1.39; 1.53 |
| 50-59 years | 2.13 | 2.09; 2.16 | 2.46 | 2.33; 2.61 | 2.01 | 1.94; 2.09 | 2.11 | 2.06; 2.16 | 2.19 | 2.11; 2.27 | 2.07 | 1.97; 2.17 |
| 60-69 years | 3.44 | 3.39; 3.49 | 4.25 | 4.01; 4.50 | 3.16 | 3.04; 3.28 | 3.47 | 3.39; 3.55 | 3.42 | 3.30; 3.55 | 3.28 | 3.12; 3.44 |
| 70-79 years | 5.28 | 5.19; 5.37 | 6.37 | 6.00; 6.76 | 4.79 | 4.60; 4.97 | 5.32 | 5.29; 5.45 | 5.36 | 5.15; 5.57 | 5.00 | 4.75; 5.25 |
| ≥ 80 years | 8.62 | 8.47; 8.76 | 9.01 | 8.44; 9.62 | 7.36 | 7.08; 7.66 | 8.99 | 8.77; 9.22 | 8.81 | 8.45; 9.19 | 8.03 | 7.61; 8.47 |
| Male (yes vs. no) | 1.24 | 1.23; 1.25 | 1.20 | 1.16; 1.24 | 1.17 | 1.14; 1.19 | 1.25 | 1.24; 1.27 | 1.28 | 1.26; 1.30 | 1.26 | 1.23; 1.29 |
| Race/color (ref: white) |  |  |  |  |  |  |  |  |  |  |  |  |
| Black | 1.06 | 1.04; 1.08 | 0.83 | 0.74; 0.92 | 0.94 | 0.89; 1.00 | 1.07 | 1.03; 1.10 | 1.07 | 1.02; 1.13 | 1.06 | 0.98; 1.14 |
| Mixed race | 0.91 | 0.89; 0.92 | 0.81 | 0.76; 0.85 | 0.77 | 0.75; 0.80 | 0.90 | 0.89; 0.91 | 1.09 | 1.05; 1.14 | 0.96 | 0.93; 0.99 |
| Asian | 0.92 | 0.89; 0.96 | 0.75 | 0.65; 0.88 | 0.69 | 0.63; 0.76 | 0.90 | 0.85; 0.95 | 1.29 | 1.15; 1.45 | 1.01 | 0.90; 1.12 |
| Indigenous | 1.05 | 0.95; 1.15 | 1.13 | 0.96; 1.33 | 0.77 | 0.60; 0.98 | 0.68 | 0.53; 0.86 | 1.16 | 0.88; 1.52 | 1.26 | 1.01; 1.58 |
| Unknown | 0.71 | 0.70; 0.72 | 0.57 | 0.52; 0.62 | 0.58 | 0.56; 0.61 | 0.65 | 0.64; 0.67 | 0.98 | 0.95; 1.02 | 0.90 | 0.87; 0.94 |
| Education (ref: no schooling/primary) |  |  |  |  |  |  |  |  |  |  |  |  |
| Lower secondary | 1.00 | 0.99; 1.02 | 0.94 | 0.88; 2.00 | 1.04 | 0.98; 1.10 | 1.04 | 1.02; 1.07 | 0.92 | 0.89; 0.96 | 0.99 | 0.93; 1.06 |
| Upper secondary | 0.95 | 0.94; 0.97 | 0.89 | 0.84; 0.94 | 0.97 | 0.93; 1.02 | 0.99 | 0.97; 1.01 | 0.88 | 0.85; 0.92 | 0.87 | 0.82; 0.92 |
| Tertiary | 0.93 | 0.91; 0.94 | 0.95 | 0.88; 1.02 | 0.93 | 0.87; 0.99 | 0.98 | 0.95; 1.01 | 0.81 | 0.77; 0.85 | 0.80 | 0.74; 0.87 |
| Unknown | 0.67 | 0.66; 0.68 | 0.55 | 0.53; 0.58 | 0.81 | 0.79; 0.84 | 0.58 | 0.57; 0.59 | 0.83 | 0.81; 0.86 | 0.99 | 0.95; 1.04 |
| Comorbidities (ref: 0) |  |  |  |  |  |  |  |  |  |  |  |  |
| 1 | 1.36 | 1.35; 1.38 | 1.22 | 1.17; 1.26 | 1.36 | 1.33; 1.39 | 1.32 | 1.30; 1.34 | 1.63 | 1.59; 1.67 | 1.38 | 1.33; 1.42 |
| 2 | 1.62 | 1.60; 1.64 | 1.37 | 1.31; 1.43 | 1.50 | 1.46; 1.54 | 1.58 | 1.56; 1.61 | 2.01 | 1.96; 2.07 | 1.73 | 1.67; 1.79 |
| ≥ 3 | 1.89 | 1.86; 1.92 | 1.41 | 1.31; 1.52 | 1.62 | 1.55; 1.68 | 1.86 | 1.82; 1.90 | 2.45 | 2.36; 2.54 | 2.08 | 1.98; 2.20 |
| Down syndrome (yes vs. no) | 1.37 | 1.27; 1.47 | 1.47 | 1.12; 1.92 | 1.47 | 1.23; 1.75 | 1.46 | 1.31; 1.62 | 1.32 | 1.12; 1.56 | 0.94 | 0.72; 1.23 |
| Obesity (yes vs. no) | 1.54 | 1.52; 1.56 | 1.82 | 1.70; 1.96 | 1.41 | 1.36; 1.46 | 1.53 | 1.50; 1.56 | 1.48 | 1.43; 1.52 | 1.69 | 1.61; 1.77 |
| Hematologic disease (yes vs. no) | 1.19 | 1.14; 1.25 | 1.03 | 0.82; 1.30 | 1.30 | 1.15; 1.46 | 1.20 | 1.13; 1.27 | 1.24 | 1.12; 1.36 | 1.01 | 0.86; 1.18 |
| Hepatic disease (yes vs. no) | 1.62 | 1.56; 1.69 | 1.39 | 1.15; 1.68 | 1.83 | 1.66; 2.01 | 1.63 | 1.54; 1.72 | 1.57 | 1.45; 1.70 | 1.52 | 1.34; 1.74 |
| Neurologic disease (yes vs. no) | 1.42 | 1.40; 1.45 | 1.12 | 1.00; 1.26 | 1.36 | 1.29; 1.43 | 1.41 | 1.37; 1.45 | 1.54 | 1.48; 1.60 | 1.28 | 1.19; 1.37 |
| Pneumopathy (yes vs. no) | 1.28 | 1.25; 1.30 | 1.40 | 1.25; 1.57 | 1.19 | 1.12; 1.26 | 1.28 | 1.25; 1.32 | 1.25 | 1.20; 1.30 | 1.29 | 1.21; 1.38 |
| Kidney disease (yes vs. no) | 1.59 | 1.56; 1.62 | 1.53 | 1.40; 1.67 | 1.70 | 1.62; 1.79 | 1.59 | 1.54; 1.63 | 1.50 | 1.44; 1.57 | 1.67 | 1.57; 1.78 |
| Immunodepression (yes vs. no) | 1.71 | 1.67; 1.75 | 1.72 | 1.54; 1/93 | 1.64 | 1.54; 1.75 | 1.69 | 1.63; 1.74 | 1.82 | 1.73; 1.91 | 1.64 | 1.51; 1.78 |
| Patient residence city’s HDI (ref: very low/Low (<0.600)) |  |  |  |  |  |  |  |  |  |  |  |  |
| Medium (0.600-0.699) | 0.96 | 0.93; 0.99 | 0.96 | 0.90; 1.03 | 0.97 | 0.94; 1.00 | 0.87 | 0.77; 0.99 | 0.59 | 0.43; 0.81 | 0.91 | 0.71; 1.17 |
| High (0.700-0.7999) | 0.81 | 0.78; 0.83 | 0.71 | 0.67; 0.77 | 0.80 | 0.77; 0.83 | 0.74 | 0.65; 0.84 | 0.53 | 0.39; 0.73 | 0.77 | 0.60; 0.99 |
| Very high (≥0.800) | 0.71 | 0.69; 0.74 | 0.54 | 0.28; 1.05 | 1.09 | 0.79; 1.52 | 0.64 | 0.56; 0.73 | 0.46 | 0.33; 0.63 | 0.73 | 0.56; 0.94 |
| Inpatient care unit’s COVID-19 hospitalizations (ref: 100-299) |  |  |  |  |  |  |  |  |  |  |  |  |
| 300-599 | 0.82 | 0.74; 0.90 | 0.65 | 0.46; 0.92 | 0.68 | 0.52; 0.89 | 0.85 | 0.74; 0.98 | 0.87 | 0.69; 1.09 | 0.91 | 0.69; 1.19 |
| 600-999 | 0.69 | 0.61; 0.77 | 1.00 | 0.61; 1.62 | 0.64 | 0.47; 0.86 | 0.64 | 0.54; 0.75 | 0.91 | 0.70; 1.18 | 0.62 | 0.44; 0.88 |
| 1000-3999 | 0.54 | 0.48; 0.60 | 0.78 | 0.47; 1,29 | 0.59 | 0.45; 0.79 | 0.45 | 0.39; 0.53 | 0.70 | 0.53; 0.92 | 0.68 | 0.51; 0.93 |
| ≥ 4000 | 0.50 | 0.36; 0.69 | 0.68 | 0.23; 2.02 | 0.39 | 0.14; 1.07 | 0.48 | 0.30; 0.76 | 0.83 | 0.38; 1.80 | 0.39 | 0.18; 0.87 |
| Healthcare unit type (ref: general hospital) |  |  |  |  |  |  |  |  |  |  |  |  |
| Specialized hospital | 0.80 | 0.66; 0.97 | 0.94 | 0.54; 1.65 | 0.72 | 0.49; 1.07 | 0.81 | 0.56; 1.17 | 0.61 | 0.33; 1.14 | 0.81 | 0.52; 1.26 |
| Mixed unit | 0.44 | 0.29; 0.66 | 0.49 | 0.16; 1.45 | 0.26 | 0.10; 0.65 | 0.51 | 0.29; 0.87 |  |  |  |  |
| General emergency center | 1.29 | 0.96; 1.72 |  |  | 1.25 | 0.55; 2.88 | 1.05 | 0.74; 1.50 |  |  | 1.49 | 0.70; 3.20 |
| Specialized emergency center | 1.44 | 0.83; 2.51 |  |  | 1.48 | 0.46; 4.77 | 1.38 | 0.69; 2.76 | 0.91 | 0.17; 4.91 |  |  |
| Country region (ref: Southeast) |  |  |  |  |  |  |  |  |  |  |  |  |
| North | 0.92 | 0.79; 1.06 |  |  |  |  |  |  |  |  |  |  |
| Northeast | 0.99 | 0.89; 1.10 |  |  |  |  |  |  |  |  |  |  |
| South | 0.78 | 0.70; 0.87 |  |  |  |  |  |  |  |  |  |  |
| Midwest | 0.95 | 0.84; 1.09 |  |  |  |  |  |  |  |  |  |  |
| Inpatient care unit out of patient’s residence city (ref.: in) | 1.06 | 1.05; 1.08 | 1.07 | 1.01; 1.14 | 1.07 | 1.04; 1.10 | 1.05 | 1.03; 1.07 | 1.04 | 1.02; 1.07 | 1.21 | 1.16; 1.27 |
| Inpatient care unit’s city size (ref: <50,000 inhabitants) |  |  |  |  |  |  |  |  |  |  |  |  |
| 50,000-99,999 inhabitants | 1.93 | 1.70; 2.20 | 1.86 | 1.21; 2.87 | 1.85 | 1.27; 2.70 | 1.94 | 1.58; 2.37 | 2.00 | 1.54; 2.59 | 1.40 | 0.94; 2.07 |
| 100,000-999,999 inhabitants | 2.79 | 2.49; 3.13 | 3.07 | 2.06; 4.57 | 2.83 | 2.00; 4.00 | 3.00 | 2.53; 3.56 | 2.19 | 1.69; 2.84 | 2.06 | 1.51; 2.82 |
| ≥ 1,000,000 inhabitants | 3.26 | 2.86; 3.71 | 4.84 | 3.06; 7.65 | 3.14 | 2.19; 4.49 | 3.12 | 2.56; 3.80 | 2.91 | 2.06; 4.11 | 2.43 | 1.72; 3.43 |
| Pandemic period (ref: Sep-Nov 2020) |  |  |  |  |  |  |  |  |  |  |  |  |
| Feb-May 2020 (wave 1.1) | 1.35 | 1.33; 1.37 | 2.10 | 1.98; 2.24 | 1.60 | 1.54; 1.67 | 1.19 | 1.16; 1.22 | 0.84 | 0.77; 0.91 | 1.40 | 1.27; 1.53 |
| Jun-Aug 2020 (wave 1.2) | 1.04 | 1.02; 1.05 | 1.13 | 1.06; 1.20 | 1.15 | 1.11; 1.19 | 0.97 | 0.95; 0.99 | 0.93 | 0.90; 0.97 | 1.31 | 1.25; 1.36 |
| Dec 2020 – Feb 2021 (wave2.1) | 1.22 | 1.20; 1.24 | 2.10 | 1.99; 2.21 | 1.28 | 1.23; 1.33 | 1.09 | 1.07; 1.11 | 1.28 | 1.24; 1.32 | 1.21 | 1.15; 1.26 |
| Mar 2021 – Apr 2021 (wave2.2) | 1.67 | 1.65; 1.69 | 2.02 | 1.90; 2.14 | 1.59 | 1.54; 1.65 | 1.60 | 1.57; 1.63 | 1.77 | 1.72; 1.82 | 1.82 | 1.75; 1.90 |
| May 2021 – Jun 2021 (wave2.3) | 1.40 | 1.38; 1.43 | 1.40 | 1.29; 1.51 | 1.32 | 1.27; 1.37 | 1.35 | 1.32; 1.38 | 1.60 | 1.55; 1.66 | 1.39 | 1.32; 1.47 |
| Jul 2021 – Dec 2021 | 0.94 | 0.92; 0.95 | 0.90 | 0.83; 0.97 | 0.96 | 0.91; 1.01 | 0.91 | 0.89; 0.93 | 0.99 | 0.96; 1.03 | 0.91 | 0.87; 0.95 |
| Jan 2022 – Feb 2022 (wave3) | 0.80 | 0.78; 0.82 | 0.70 | 0.64; 0.76 | 0.82 | 0.78; 0.86 | 0.82 | 0.80; 0.85 | 0.76 | 0.73; 0.80 | 0.74 | 0.69; 0.79 |
| Mar 2022 – Dec 2022 | 0.52 | 0.51; 0.53 | 0.46 | 0.41; 0.52 | 0.55 | 0.52; 0.58 | 0.49 | 0.48; 0.51 | 0.56 | 0.54; 0.59 | 0.50 | 0.47; 0.54 |

Source: SIVEP Gripe – Sistema de Informação de Vigilância Epidemiológica da Gripe.

The study excluded inpatient care units with less than 100 COVID-19 hospitalizations in the period.

**Table S3.3. Distribution of individuals by race/color in Brazil and macro-regions**

| Race/color | Brazil | | North | | Northeast | | Southeast | | South | | Midwest | |
| --- | --- | --- | --- | --- | --- | --- | --- | --- | --- | --- | --- | --- |
|  | N | % | N | % | N | % | N | % | N | % | N | % |
| White | 713,807 | 44.2 | 8,935 | 9.0 | 27,616 | 11.2 | 399,810 | 49.8 | 241,042 | 79.8 | 36,404 | 22.3 |
| Black | 64,697 | 4.0 | 2,675 | 2.7 | 8,624 | 3.5 | 40,236 | 5.0 | 8,251 | 2.7 | 4,911 | 3.0 |
| Mixed-race | 518,786 | 32.1 | 78,888 | 79.4 | 140,946 | 57.0 | 204,256 | 25.4 | 18,571 | 6.2 | 76,125 | 46.6 |
| Asian | 15,367 | 0.9 | 1,091 | 1.1 | 2,634 | 1.1 | 8,004 | 1.0 | 1,622 | 0.5 | 2,016 | 1.2 |
| Indigenous | 2,528 | 0.2 | 979 | 1.0 | 338 | 0.1 | 433 | 0.1 | 329 | 0.1 | 449 | 0.3 |
| Unknown | 300,243 | 18.6 | 6,797 | 6.8 | 67,084 | 27.1 | 150,859 | 18.8 | 32,181 | 10.7 | 43,322 | 26.5 |

Source: SIVEP Gripe – Sistema de Informação de Vigilância Epidemiológica da Gripe.

**Table S3.4. Distribution of individuals by education level in Brazil and macro-regions**

|  | Brazil | | North | | Northeast | | Southeast | | South | | Midwest | |
| --- | --- | --- | --- | --- | --- | --- | --- | --- | --- | --- | --- | --- |
|  | N | % | N | % | N | % | N | % | N | % | N | % |
| No schooling | 34,439 | 2.1 | 5,355 | 5.4 | 8,497 | 3.4 | 13,302 | 1.7 | 4,543 | 1.5 | 2,742 | 1.7 |
| Primary | 153,134 | 9.5 | 12,100 | 12.2 | 15,962 | 6.5 | 77,149 | 9.6 | 36,773 | 12.2 | 11,150 | 6.8 |
| Lower secondary | 109,540 | 6.8 | 8,325 | 8.4 | 9,652 | 3.9 | 53,425 | 6.7 | 28,963 | 9.6 | 9,175 | 5.6 |
| Upper secondary | 190,215 | 11.8 | 15,759 | 15.9 | 18,417 | 7.5 | 100,113 | 12.5 | 40,470 | 13.4 | 15,456 | 9.5 |
| Tertiary | 92,573 | 5.7 | 6,670 | 6.7 | 8,856 | 3.6 | 50,186 | 6.3 | 18,798 | 6.2 | 8,063 | 4.9 |
| Unknown | 1,035,527 | 64.1 | 51,156 | 51.5 | 185,858 | 75.2 | 509,423 | 63.4 | 172,449 | 57.1 | 116,641 | 71.5 |

Source: SIVEP Gripe – Sistema de Informação de Vigilância Epidemiológica da Gripe.

**Table S3.5. Distribution of known and unknown data on race/color and education level by inpatient care unit categories in Brazil and macro-regions**

| Region/ Variable | Public  SUS | | Public non-SUS | | Private  SUS | | Private non-SUS | | Philanthropic SUS | | Philanthropic non-SUS | | Total | |
| --- | --- | --- | --- | --- | --- | --- | --- | --- | --- | --- | --- | --- | --- | --- |
|  | N | % | N | % | N | % | N | % | N | % | N | % | N | % |
| **Brazil** |  |  |  |  |  |  |  |  |  |  |  |  |  |  |
| Total | 632,024 | 39.1 | 13,599 | 0.8 | 70,175 | 4.3 | 346,035 | 21.4 | 475,456 | 29.4 | 78,139 | 4.8 | 1,615,428 | 100.0 |
| Race/Color |  |  |  |  |  |  |  |  |  |  |  |  |  |  |
| Known | 530,931 | 84.0 | 8,277 | 60.9 | 55,365 | 78.9 | 234,065 | 67.6 | 432,530 | 91.0 | 54,017 | 69.1 | 1,315.185 | 81.4 |
| Unknown | 101,093 | 16.0 | 5,322 | 39.1 | 14,810 | 21.1 | 111,970 | 32.4 | 42,926 | 9.0 | 24,122 | 30.9 | 300,243 | 18.6 |
| Education |  |  |  |  |  |  |  |  |  |  |  |  |  |  |
| Known | 209,912 | 33.2 | 5,105 | 37.5 | 26,020 | 37.1 | 107,517 | 31.1 | 209,222 | 44.0 | 22,125 | 28.3 | 579,901 | 35.9 |
| Unknown | 422,112 | 66.8 | 8,494 | 62.5 | 44,155 | 62.9 | 238,518 | 68.9 | 266,234 | 56.0 | 56,014 | 71.7 | 1,035,527 | 64,1 |
| **North** | | | | | | | | | | | | | | |
| Total | 78,388 | 78.9 | 508 | 0.5 | 3,151 | 3.2 | 8,447 | 8.5 | 4,735 | 4.8 | 4,136 | 4.2 | 99,365 | 100.0 |
| Race/Color |  |  |  |  |  |  |  |  |  |  |  |  |  |  |
| Known | 74,833 | 95.5 | 408 | 80.3 | 2,982 | 94.6 | 6,377 | 75.5 | 4,495 | 94.9 | 3,473 | 84.0 | 92,568 | 93.2 |
| Unknown | 3,555 | 4.5 | 100 | 19.7 | 169 | 5.4 | 2,070 | 24.5 | 240 | 5.1 | 663 | 16.0 | 6.797 | 6.8 |
| Education |  |  |  |  |  |  |  |  |  |  |  |  |  |  |
| Known | 39,163 | 50.0 | 248 | 48.8 | 1,992 | 63.2 | 3,192 | 37.8 | 1,844 | 38.9 | 1,770 | 42.8 | 48,209 | 48.5 |
| Unknown | 39,225 | 50.0 | 260 | 51.2 | 1,159 | 36.8 | 5,255 | 62.2 | 2,891 | 61.1 | 2366 | 57.2 | 51,156 | 51.5 |
| **Northeast** | | | | | | | | | | | | | | |
| Total | 148,839 | 60.2 | 1,058 | 0.4 | 12,761 | 5.2 | 50,965 | 20.6 | 29,981 | 12.1 | 3,638 | 1.5 | 247,242 | 100.0 |
| Race/Color |  |  |  |  |  |  |  |  |  |  |  |  |  |  |
| Known | 117,058 | 78.7 | 802 | 75.8 | 9,484 | 74.3 | 25,412 | 49.9 | 24,031 | 80.2 | 3371 | 92.7 | 180,158 | 72.9 |
| Unknown | 31,781 | 21.3 | 256 | 24.2 | 3,277 | 25.7 | 25,553 | 50.1 | 5,950 | 19.8 | 267 | 7.3 | 67,084 | 27.1 |
| Education |  |  |  |  |  |  |  |  |  |  |  |  |  |  |
| Known | 40,681 | 27.3 | 305 | 28.8 | 2,265 | 17.7 | 10,406 | 20.4 | 7,184 | 24.0 | 543 | 14.9 | 61,384 | 24.8 |
| Unknown | 108,158 | 72.7 | 753 | 71.2 | 10,496 | 82.3 | 40,559 | 79.6 | 22,797 | 76.0 | 3,095 | 85.1 | 185,858 | 75.2 |
| **Southeast** | | | | | | | | | | | | | | |
| Total | 253,990 | 31.6 | 9,613 | 1.2 | 13,711 | 1.7 | 217,564 | 27.1 | 256,557 | 31.9 | 52,163 | 6.5 | 803598 | 100.0 |
| Race/Color |  |  |  |  |  |  |  |  |  |  |  |  |  |  |
| Known | 215,108 | 84.7 | 5,428 | 56.5 | 11,204 | 81.7 | 153,380 | 70.5 | 235704 | 91.9 | 31,915 | 61.2 | 652,739 | 81.2 |
| Unknown | 38,882 | 15.3 | 4,185 | 43.5 | 2,507 | 18.3 | 64,184 | 29.5 | 20,853 | 8.1 | 20,248 | 38.8 | 150,859 | 18.8 |
| Education |  |  |  |  |  |  |  |  |  |  |  |  |  |  |
| Known | 83,292 | 32.8 | 4,299 | 44.7 | 5,411 | 39.5 | 71,224 | 32.7 | 117,560 | 45.8 | 12,389 | 23.8 | 294,175 | 36.6 |
| Unknown | 170,698 | 67.2 | 5314 | 55.3 | 8,300 | 60.5 | 146,340 | 67.3 | 138997 | 54.2 | 39,774 | 76.2 | 509,423 | 63.4 |
| **South** | | | | | | | | | | | | | | |
| Total | 68,235 | 22.6 | 928 | 0.3 | 18,445 | 6.1 | 34,069 | 11.3 | 165,912 | 54.9 | 14,407 | 4.8 | 301,996 | 100.0 |
| Race/Color |  |  |  |  |  |  |  |  |  |  |  |  |  |  |
| Known | 60,121 | 88.1 | 858 | 92.5 | 16,990 | 92.1 | 26,165 | 76.8 | 153,115 | 92.3 | 12,566 | 87.2 | 269,815 | 89.3 |
| Unknown | 8,114 | 11.9 | 70 | 7.5 | 1,455 | 7.9 | 7,904 | 23.2 | 12,797 | 7.7 | 1,841 | 12.8 | 32,181 | 10.7 |
| Education |  |  |  |  |  |  |  |  |  |  |  |  |  |  |
| Known | 21,389 | 31.3 | 75 | 8.1 | 9,311 | 50.5 | 15,990 | 46.9 | 77,116 | 46.5 | 5,666 | 39.3 | 129,547 | 42.9 |
| Unknown | 46,846 | 68.7 | 853 | 91.9 | 9,134 | 49.5 | 18079 | 53.1 | 88,796 | 53.5 | 8,741 | 60.7 | 172,449 | 57.1 |
| **Midwest** |  |  |  |  |  |  |  |  |  |  |  |  |  |  |
| Total | 82,572 | 50.6 | 1,492 | 0.9 | 22,107 | 13.5 | 34,990 | 21.4 | 18,271 | 11.2 | 3795 | 2.3 | 163,227 | 100.0 |
| Race/Color |  |  |  |  |  |  |  |  |  |  |  |  |  |  |
| Known | 63,811 | 77.3 | 781 | 52.4 | 14,705 | 66.5 | 22,731 | 65.0 | 15,185 | 83.1 | 2,692 | 70.9 | 119,905 | 73.5 |
| Unknown | 18761 | 22.7 | 711 | 47.6 | 7,402 | 33.5 | 12,259 | 35.0 | 3,086 | 16.9 | 1,103 | 29.1 | 43,322 | 26.5 |
| Education |  |  |  |  |  |  |  |  |  |  |  |  |  |  |
| Known | 25,387 | 30.8 | 178 | 11.9 | 7,041 | 31.8 | 6,705 | 19.2 | 5,518 | 30.2 | 1,757 | 46.3 | 46,586 | 28.5 |
| Unknown | 57,185 | 69.2 | 1,314 | 88.1 | 15,066 | 68.2 | 28,285 | 80.8 | 12,753 | 69.8 | 2,038 | 53.7 | 116,641 | 71.5 |
