# Supplementary material for: COVID-19 inpatient mortality in Brazil from 2020 to 2022: a cross-sectional overview study based on secondary data
Source: Int J Equity Health. 2023 Nov 17;22:238. doi: 10.1186/s12939-023-02037-8 (PMC10655483; doi:10.1186/s12939-023-02037-8)
Supplement: Supplementary file 4 — Additional file 4: Supplement 4. Generalized linear mixed models: factors associated with COVID-19 inpatient mortality in Southern Brazil, Feb 2020 – Dec 2022. [file 12939_2023_2037_MOESM4_ESM.docx]

Supplement 4. Generalized linear mixed models: factors associated with COVID-19 inpatient mortality in Southern Brazil, Feb 2020 – Dec 2022.

| Variable | South | |
| --- | --- | --- |
|  | OR | 95%CI |
| Inpatient healthcare unit category (ref: Public SUS) |  |  |
| Public non-SUS | 0.65 | 0.34; 1.23 |
| Private SUS | 1.00 | 0.66; 1.52 |
| Private non-SUS | 0.60 | 0.45; 0.81 |
| Philanthropic SUS | 1.20 | 0.97; 1.48 |
| Philanthropic non-SUS | 0.38 | 0.24; 0.60 |
| Age (ref: 18-39 years) |  |  |
| 40-49 years | 1.48 | 1.41; 1.56 |
| 50-59 years | 2.18 | 2.09; 2.28 |
| 60-69 years | 3.64 | 3.48; 3.81 |
| 70-79 years | 6.63 | 6.34; 6.95 |
| ≥ 80 years | 15.24 | 14.51; 16.00 |
| Male (yes vs. no) | 1.22 | 1.20; 1.25 |
| Race/color (ref: white/unknown) |  |  |
| Black | 1.10 | 1.03; 1.17 |
| Mixed race | 1.12 | 1.07; 1.18 |
| Asian | 1.27 | 1.10; 1.45 |
| Indigenous | 1.17 | 0.85; 1.62 |
| Comorbidities (ref: 0) |  |  |
| 1 | 1.56 | 1.51; 1.60 |
| 2 | 1.85 | 1.79; 1.91 |
| ≥ 3 | 2.22 | 2.13; 2.32 |
| Down syndrome (yes vs. no) | 1.12 | 0.92; 1.35 |
| Obesity (yes vs. no) | 1.04 | 1.00; 1.08 |
| Hematologic disease (yes vs. no) | 1.32 | 1.18; 1.48 |
| Hepatic disease (yes vs. no) | 1.67 | 1.51; 1.84 |
| Neurologic disease (yes vs. no) | 1.91 | 1.83; 2.00 |
| Pneumopathy (yes vs. no) | 1.25 | 1.19; 1.30 |
| Kidney disease (yes vs. no) | 1.51 | 1.43; 1.59 |
| Immunodepression (yes vs. no) | 2.25 | 2.12; 2.39 |
| ICU use (yes vs. no) | 4.20 | 4.09; 4.32 |
| Ventilatory support use (ref.: no) |  |  |
| Invasive | 11.37 | 10.92; 11.85 |
| Non-invasive | 1.64 | 1.58; 1.69 |
| Length of stay (ref: ≥ 1 day) |  |  |
| 0 day | 4.24 | 3.92; 4.59 |
| Unknown | 0.20 | 0.18; 0.23 |
| Patient residence city’s HDI (ref: very low/low (<0.600)) |  |  |
| Medium (0.600-0.699) | 0.54 | 0.37; 0.79 |
| High (0.700-0.7999) | 0.51 | 0.35; 0.76 |
| Very high (≥0.800) | 0.45 | 0.31; 0.66 |
| Inpatient care unit’s COVID-19 hospitalizations ref: 100-299) |  |  |
| 300-599 | 0.70 | 0.57; 0.86 |
| 600-999 | 0.59 | 0.47; 0.74 |
| 1000-3999 | 0.50 | 0.39; 0.63 |
| ≥ 4000 | 0.60 | 0.31; 1.18 |
| Healthcare unit type (ref: general hospital) |  |  |
| Specialized hospital | 0.71 | 0.41; 1.23 |
| Specialized emergency center | 0.59 | 0.13; 2.65 |
| Inpatient care unit out of patient’s residence city (ref.: in) | 0.99 | 0.96; 1.02 |
| Inpatient care unit’s city size (ref: <50,000 inhabitants) |  |  |
| 50,000-99,999 inhabitants | 1.52 | 1.21; 1.90 |
| 100,000-999,999 inhabitants | 1.54 | 1.23; 1.93 |
| ≥ 1,000,000 inhabitants | 1.75 | 1.29; 2.36 |
| Pandemic period (ref: Sep-Nov 2020) |  |  |
| Feb-May 2020 (wave 1.1) | 0.73 | 0.66; 0.81 |
| Jun-Aug 2020 (wave 1.2) | 0.94 | 0.90; 0.98 |
| Dec 2020 – Feb 2021 (wave2.1) | 1.34 | 1.29; 1.39 |
| Mar 2021 – Apr 2021 (wave2.2) | 1.77 | 1.71; 1.83 |
| May 2021 – Jun 2021 (wave2.3) | 1.49 | 1.42; 1.55 |
| Jul 2021 – Dec 2021 | 0.91 | 0.87; 0.94 |
| Jan 2022 – Feb 2022 (wave3) | 0.83 | 0.79; 0.88 |
| Mar 2022 – Dec 2022 | 0.65 | 0.61; 0.68 |

Source: SIVEP Gripe - Sistema de Informação de Vigilância Epidemiológica da Gripe.

The study excluded inpatient care units with less than 100 COVID-19 hospitalizations in the period.
